# HECTD1 promotes base excision repair in nucleosomes through chromatin remodelling

**DOI:** 10.1093/nar/gkz1129

**Published:** 2019-12-04

**Authors:** Laura Bennett, Eleanor C E T Madders, Jason L Parsons

**Affiliations:** Cancer Research Centre, Department of Molecular and Clinical Cancer Medicine, University of Liverpool, 200 London Road, Liverpool L3 9TA, UK

## Abstract

Base excision repair (BER) is the major cellular DNA repair pathway that recognises and excises damaged DNA bases to help maintain genome stability. Whilst the major enzymes and mechanisms co-ordinating BER are well known, the process of BER in chromatin where DNA is compacted with histones, remains unclear. Using reconstituted mononucleosomes containing a site-specific synthetic abasic site (tetrahydrofuran, THF), we demonstrate that the DNA damage is less efficiently incised by recombinant AP endonuclease 1 (APE1) when the DNA backbone is facing the histone core (THF-in) compared to that orientated away (THF-out). However, when utilizing HeLa whole cell extracts, the difference in incision of THF-in versus THF-out is less pronounced suggesting the presence of chromatin remodelling factors that stimulate THF accessibility to APE1. We subsequently purified an activity from HeLa cell extracts and identify this as the E3 ubiquitin ligase, HECTD1. We demonstrate that a recombinant truncated form of HECTD1 can stimulate incision of THF-in by APE1 *in vitro* by histone ubiquitylation, and that siRNA-mediated depletion of HECTD1 leads to deficiencies in DNA damage repair and decreased cell survival following x-ray irradiation, particularly in normal fibroblasts. Thus, we have now identified HECTD1 as an important factor in promoting BER in chromatin.

## INTRODUCTION

Reactive oxygen species (ROS) that are generated endogenouslythrough cellular oxidative metabolism, but also by exogenous sources such as ionizing radiation and environmental toxins, cause a constant bombardment on our cellular DNA. As a result, ROS can directly react with the DNA molecule forming DNA base oxidation, base loss (apurinic/apyrimidinic or AP sites) and DNA single and double strand breaks (SSB and DSB). If the DNA damage is left unrepaired, this can cause mutations and ultimately has been linked to premature ageing, age-related neurodegenerative diseases such as Alzheimer's and Parkinson's, and cancer. Remarkably, as a consequence of cellular metabolism ∼10 000 DNA base damage events occur in every human cell per day (1). These are usually corrected and repaired in cells by the base excision repair (BER) pathway, which is dedicated to excising damaged DNA bases and replacing these with the correct undamaged nucleotides (2,3). This pathway also repairs AP sites and SSBs and plays a vital role in maintaining genome stability through suppressing DNA damage accumulation, and in the prevention of human disease development. Indeed, BER performs a vital role in normal development and survival since knockout mouse models, particularly of downstream factors involved in BER, display an embryonic lethal phenotype. Genome instability and an increase in sensitivity to DNA damaging agents is furthermore evident following siRNA-mediated knockdowns of key BER proteins in cultured cells (4–7), further highlighting that BER is an essential DNA repair process required for normal cellular functioning.

BER is achieved in a co-ordinated manner by a specific subset of enzymes. In the first step, the damaged DNA bases are excised by damage specific DNA glycosylases, of which 11 human enzymes are currently known to exist. Generally, this creates an AP site which is recognised and incised by AP endonuclease-1 (APE1). DNA polymerase β (Pol β) then removes the 5′-deoxyribose phosphate (5′-dRP) moiety, inserts the correct nucleotide into the repair gap and DNA ligase IIIα-X-ray cross complementing protein 1 (Lig IIIα-XRCC1) complex seals the DNA ends to complete repair. Despite this knowledge of the BER process, little is understood about the mechanism of action in chromatin. The building blocks of chromatin are nucleosomes, which consist of ∼146 bp of DNA wrapped around a histone octamer containing the histone proteins H2A, H2B, H3 and H4 (two of each). In order for the cell to undergo DNA-dependent activities, such as transcription and replication, the chromatin structure has to be altered to enable enzyme accessibility. This process is achieved by ATP-dependent chromatin remodelling factors (8). However post-translational modifications, including acetylation, phosphorylation and ubiquitylation, on the N-terminal tails of the histones aid to recruit these enzymes as well as to stimulate chromatin decondensation. Evidence suggests that DNA repair also requires the induction of histone modifications, particularly ubiquitylation (9), and that chromatin remodellers are necessary to improve DNA damage accessibility and ensure an efficient DNA repair process. Most of the evidence, however, has been centred around the recognition and repair of DNA DSBs (10), in which ATM-dependent phosphorylation of H2AX and ubiquitylation of H2A and γH2AX catalysed by the E3 ubiquitin ligases RNF8 and RNF168 are known to play prominent roles. Chromatin remodellers including p400, NuRD and ALC1 are then thought to promote relaxation of chromatin and allow accessibility of the DSB to DNA repair enzymes (11–13).

Specifically related to BER repair in chromatin, there is now building evidence from *in vitro* biochemical assays employing reconstituted mononucleosome substrates containing DNA damage at specific sites, that the activities of recombinant BER proteins are retarded or inhibited by chromatin structure. This appears to be dependent on the position and orientation of the damage relative to the nucleosome. For example, DNA glycosylases including uracil DNA glycosylase (UDG) and endonuclease III homologue (NTH1) can efficiently excise uracil and oxidative DNA damage, respectively when the DNA backbone is outwardly facing from the nucleosome, but are inhibited when the DNA backbone is inwardly facing (14,15). Likewise, APE1 displays orientation-dependent differences in activities versus AP sites in mononucleosomes (16). Repair of a one nucleotide gap by Pol β (17) and of a SSB by XRCC1-DNA ligase IIIα (18) is also severely impacted when the damage is placed within a mononucleosome. Therefore, chromatin structure greatly impacts the efficiency of BER by preventing DNA damage accessibility and processing. Collectively, these studies have highlighted that chromatin decompaction or remodelling is required, particularly for sterically occluded DNA base damage in chromatin. Indeed, there is evidence that purified SWI/SNF and RSC can increase the activities of BER proteins on mononucleosome substrates (19,20), and very recently FACT has also been shown to assist in BER (21). Additionally, other factors have been shown to be present in human cells that stimulate DNA glycosylase activity on mononucleosomes, but these have not been specifically identified (22). Therefore to date, the identity of specific histone modifiers and chromatin remodelling enzymes stimulating BER in chromatin *in vivo* is unclear.

In this study, we demonstrate that a synthetic AP site (tetrahydrofuran, THF) with the backbone facing inwards towards the histone core is refractory to incision by recombinant APE1, but can be more efficiently incised by APE1 present within human whole cell extracts. We have subsequently isolated and purified enzymatic activities that stimulate APE1 activity on a mononucleosome substrate, and now describe the E3 ubiquitin ligase HECTD1 as a new player in the BER pathway which acts to promote histone ubiquitylation and therefore increase DNA damage accessibility within chromatin.

## MATERIALS AND METHODS

### DNA, oligonucleotides and plasmids

The pGEM-3Z-601 plasmid, *Xenopus laevis* histone (H2A, H2B, H3 and H4) bacterial expression plasmids, the bacterial APE1 expression plasmid and the mammalian expression plasmid for full length murine HECTD1 were kindly provided by P. O’Neill, Prof K. Luger, Prof. G. Dianov and Prof. I. Zohn, respectively. The C-terminal cDNA sequence of murine *hectd1* (amino acids 1762–2612) containing the active E3 ligase HECT domain was recloned into pET28a by ligation-independent cloning (23) to enable expression of truncated murine HECTD1 protein (ΔN-HECTD1). Site directed mutagenesis was used to generate the inactive E3 ligase mutant (C2579G) of the truncated HECTD1 protein (ΔN-mutHECTD1). Bacterial expression plasmids for E1 conjugating enzyme (UBE1) and E2 conjugating enzymes (UbE2H, Cdc34a, UbE2D1, UbE2D2, UbE2D3, UbE1E1, UbE2L3, UbE2L6 and UbE2C) were acquired from Addgene (Teddington, UK). Recombinant ubiquitin was purchased from Boston Biochemicals (Cambridge, USA). IRDye 700/800–5′-labelled primers used to PCR amplify the 256 bp 601 DNA, and oligonucleotides containing the synthetic AP site tetrahydrofuran (THF), were from IDT Technologies (Leuven, Belgium).

### Purification of recombinant proteins

Histone proteins were expressed in Rosetta2(DE3)pLysS bacterial cells (Merck-Millipore, Watford, UK) and purified under denaturing conditions similar to that previously described (24). In brief, individual histone proteins were isolated from inclusion bodies from 500 ml bacterial cultures and purified using a HiPrep 26/60 Sephacryl S-200 HR column (GE Healthcare, Little Chalfont, UK) in separation buffer (20 mM sodium acetate, pH 5.2, 7 M urea, 1 mM EDTA and 5 mM β-mercaptoethanol) containing 1 M NaCl. Fractions containing the histones were identified using 16% SDS-PAGE and Instant Blue (Expedeon Ltd, Over, UK) staining and dialysed overnight in water containing 2 mM β-mercaptoethanol. Histones were then added to a Mono-S 5/50 GL column (GE Healthcare, Little Chalfont, UK) in separation buffer containing 0.1 M NaCl, and proteins eluted using a 20 ml linear gradient with buffer containing 1 M NaCl. Fractions containing the histones were similarly identified using 16% SDS-PAGE and Instant Blue staining and dialysed in water containing 2 mM β-mercaptoethanol. Histones were concentrated using Amicon Ultra-15 centrifugal filter units (Millipore, Watford, UK), protein concentrations measured using a Nanodrop ND-1000 spectrometer (Thermo Fisher Scientific, Runcorn, UK) at a wavelength of OD 280 nm and aliquots of H2A (2 mg), H2B (2 mg), H3 (2.25 mg) and H4 (1.75 mg) frozen at −80°C. The his-tagged proteins ΔN-HECTD1, ΔN-mutHECTD1, APE1, E1 activating enzyme and E2 conjugating enzymes were overexpressed in Rosetta2(DE3)pLysS bacterial cells and purified using HisTrap column chromatography (GE Healthcare, Little Chalfont, UK) using a gradient elution of imidazole and an AKTA purifier FPLC system. Fractions containing purified proteins were concentrated and buffer exchanged using Amicon Ultra-15 centrifugal filters into storage buffer (25 mM Tris–HCl, pH 8.0, 100 mM KCl, 12 MgCl_2_, 1 mM EDTA, 17% glycerol and 1 mM DTT) prior to aliquoting and storage at −80°C.

### Preparation of histone octamer

Each histone aliquot of H2A, H2B, H3 and H4 was dissolved to 2 mg/ml in unfolding buffer (20 mM Tris–HCl, pH 7.5, 7 M guanidinium HCl, 10 mM DTT) on ice for 2 h, proteins were mixed together and a 1 mg/ml solution created. Histones were dialysed in snakeskin dialysis tubing (Thermo Scientific, Runcorn, UK) in refolding buffer (10 mM Tris–HCl, pH 7.5, 2 M NaCl, 1 mM EDTA and 5 mM β-mercaptoethanol) for 6 h at 4°C, overnight and then for 4 h in fresh buffer. The dialysed histone octamer was centrifuged at 23 000 × g for 20 min at 4°C, the supernatant collected and concentrated using Amicon Ultra-15 centrifugal filters to ∼400 μl. The octamer was loaded in two batches (200 μl each) onto a Superdex 200 10/300 GL gel filtration column (GE Healthcare, Little Chalfont, UK) in refolding buffer. Fractions were analysed using 16% SDS-PAGE and Instant blue staining, those containing the histone octamer in the correct equimolar ratios were pooled and concentrated using Amicon Ultra-15 centrifugal concentrators to ∼10 mg/ml, glycerol was added and the histone octamer stored at −20°C.

### Preparation of DNA containing site-specific DNA damage

**Figure 1.**
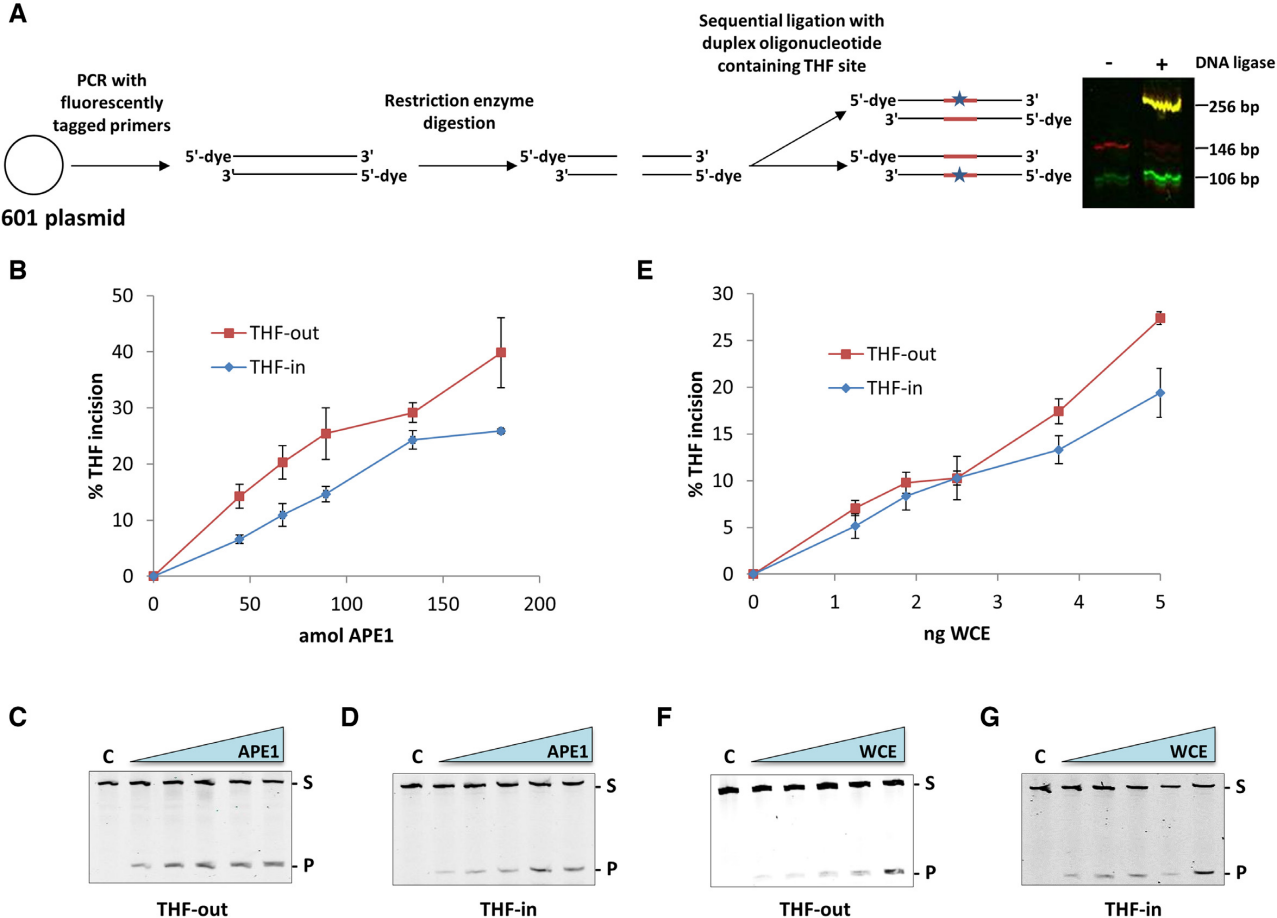
The incision of free DNA oligonucleotide substrates containing THF in two different orientations is similar by recombinant APE1 and HeLa WCE. (**A**) The 601 DNA sequence was amplified by PCR using primers containing either a 5′-IR Dye 700 or 5′-IR Dye 800 termini, a central region was removed by restriction enzyme digestion and replaced by a two-step ligation with a duplex oligonucleotide containing THF on the upper strand (THF-out) or lower strand (THF-in). Full length 256 bp products from the final ligation (see representative figure) were separated and purified by non-denaturing PAGE. Incision of free THF-out and THF-in substrates (50 fmol) by increasing amounts of recombinant APE1 (**B**–**D**) and (**E**–**G**) HeLa WCE incubated for 10 min at 30°C. Shown is the mean percent substrate incision ± S.D. from at least three independent experiments, and also representative images from the respective gels.

The DNA substrates were prepared as previously described (25), but with modifications (see also Figure 1A). The 601 DNA sequence was amplified from the pGEM-3Z-601 plasmid using the following primers (5′-IRDye 700-GCTCGGAATTCTATCCGACTGGCACCGGCAAG-3′ and 5′-IRDye 800-GCATGATTCTTAAGACCGAGTTCATCCCTTATGTG-3′). The 256 bp DNA was purified using the QIAquick PCR purification kit (Qiagen, Manchester, UK) and the central 20 bp region released following digestion with Van91I and BglI (Thermo Fisher Scientific, Runcorn, UK) overnight at 37°C. The two fragments (127 and 106 bp) were separated by 8% non-denaturing PAGE, located using the Odyssey Image Analysis System (LiCor Biosciences, Cambridge, UK), excised and purified from the gel pieces by freezing these at −80°C, incubating them in TE at 37°C for 3 h, and then concentrating the DNA using Amicon Ultra-0.5 centrifugal filters. Oligonucleotides containing THF in one of two positions (5′-phosphate-TTGGTGCXTTTAAGCCGTGC-3′ and 5′-phosphate-CGGCTTAAAYGCACCAACGC-3′; where X is equivalent to the DNA backbone orientated outwardly facing away from the nucleosome (THF-out) and Y with the DNA backbone orientated inwardly facing towards the nucleosome (THF-in) were annealed in TE buffer containing 200 mM NaCl by heating at 95°C for 5 min and slow cooling to room temperature. The duplex oligonucleotide (1.5-fold excess) containing the site-specific THF was then annealed firstly to the 127 bp DNA using T4 DNA ligase (Thermo Fisher Scientific, Runcorn, UK) in ∼60 μl overnight at 4°C, purified using the MinElute Reaction Cleanup Kit (Qiagen, Manchester, UK), and then a second ligation reaction containing ∼1.5-fold of the 106 bp DNA was performed in ∼80 μl overnight at 4°C. The 256 bp product was separated by 8% non-denaturing PAGE and purified from the excised gel pieces as described above.

### Nucleosome reconstitution

Nucleosomes were reconstituted using salt dialysis. The 5′-IRDye700/800-labelled 256 bp DNA containing the site-specific THF site (5 pmol) was mixed with unlabelled 256 bp 601 DNA (75 pmol) and equimolar of purified histone octamer (80 pmol) in 50 μg BSA, 2 M NaCl and 0.01% NP-40 in a volume of ∼300 μl. This was then dialysed in 500 ml dialysis buffer (10 mM Tris–HCl, pH 7.4, 1 mM EDTA, 5 mM β-mercaptoethanol) containing 1.6 M NaCl for 1.5 h. The buffer was then sequentially replaced with dialysis buffer containing 1.2, 0.8, 0.6 and 0.2 M NaCl for 1.5 h each, and then with dialysis buffer containing 75 mM NaCl overnight. The efficiency of nucleosome reconstitution was examined on a 0.7% agarose gel containing SYTO-60 using the Odyssey Image Analysis System to ensure at least 95% complete before it was acceptable for use in *in vitro* assays.

### *In vitro* BER assays

Reactions (10 μl) contained 50 fmol free or mononucleosomal DNA containing the site-specific THF site, 0.7 pmol GST-E1 activating enzyme, 2.5 pmol E2 conjugating enzyme (combination of 9 different E2s), 0.6 nmol ubiquitin (Boston Biochemicals, Cambridge, USA) and 1 μg acetylated BSA in buffer containing 25 mM Tris-HCl (pH 8.0), 50 mM KCl, 2 mM ATP, 8.5 mM MgCl_2_, 0.5 mM EDTA, 8.5% glycerol and 1 mM DTT. These were incubated in LoBind protein tubes (Eppendorf, Stevenage, UK) for 1 h at 30°C, unless otherwise indicated, with agitation. Reactions were stopped by the addition of 20 mM EDTA and 0.4% SDS and the DNA extracted using phenol:chloroform:isoamyl alcohol (25:24:1) and then twice with chloroform:isoamyl alcohol (24:1). The DNA was precipitated using 2.5 volumes ice-cold ethanol in the presence of 10 μg glycogen and 0.3 M sodium acetate (pH 5.2) at −80°C for 1 h. The precipitated DNA was resuspended thoroughly in 10 μl TE buffer, and then 10 μl formamide loading dye (95% formamide, 2.5 mg/ml bromophenol blue) added. Samples were heated for 5 min at 95°C prior to analysis by 8% denaturing PAGE (7 M urea) and substrate incision quantified using the Odyssey Image Analysis System.

### Whole cell extract preparation, fractionation and immunoblotting

Whole cell extracts (WCE) were prepared as previously described (26,27). WCE from 20 g HeLa cell pellets (Cilbiotech, Belgium) was fractionated using column chromatography, and proteins present in active fractions from the final Mono-Q chromatography were identified by tandem mass spectrometry, as recently described (27,28). Following each chromatography stage, protein fractions were analysed for *in vitro* BER activity using a free or mononucleosome substrate and active fractions pooled for the next chromatography step. Immunoblotting was performed as described in the references above, using the Odyssey Image Analysis System for protein detection and quantification. Primary antibodies raised against APE1 were kindly provided by Prof. G.Dianov, HECTD1 and Mule antibodies were from Bethyl Laboratories (Montgomery, USA), tubulin antibodies were from Sigma (Dorset, UK), Cul4A, DDB1 and histone H2B and H4 antibodies were from Abcam (Cambridge, UK), and histone H3 and H4 antibodies were from Cell Signaling (London, UK).

### Cell culture and RNA interference

HeLa cells or normal human lung fibroblasts (AG06173 or WI-38) were cultured in Dulbecco's modified Eagle's medium (DMEM) supplemented with 10% fetal bovine serum, 2 mM l-glutamine, 1× penicillin–streptomycin and 1× non-essential amino acids at 37°C in 5% CO_2_. siRNA knockdown of HECTD1 (SMARTpool siGENOME; GE Healthcare, Little Chalfont, UK) or using a non-targeting control siRNA (AllStars Negative Control siRNA; Qiagen, Manchester, UK) were performed using Lipofectamine RNAiMAX (Life Technologies, Paisley, UK) for 48 h.

### **Single cell gel electrophoresis (Comet) and clonogenic assays**.

The alkaline comet assay for measurement of DNA single strand breaks and alkali labile sites, as well as clonogenic survival assays, were performed as recently described (27,29). For comets, cells (50 per slide, in duplicate) were analysed using the Komet 6.0 image analysis software (Andor Technology, Belfast, Northern Ireland) and % tail DNA values averaged from at least three independent experiments. For clonogenic assays, colonies were counted using the GelCount colony analyser (Oxford Optronics, Oxford, UK), and surviving fraction was expressed as colonies per treatment level versus colonies that appeared in the untreated control.

## RESULTS

### THF-in mononucleosome substrate is inefficiently incised by recombinant APE1

An increasing number of reports have demonstrated that the efficiency of excision of DNA base damage by purified BER proteins within a mononucleosome substrate is dependent on positioning relative to the histone core, and predicted that BER requires chromatin remodelling to stimulate repair of DNA base damage in sterically occluded regions. However, a comparison of substrate cleavage by recombinant BER proteins versus proteins present within cell extracts where factors promoting histone modifications and chromatin remodelling are present, has not been thoroughly analysed. We generated two fluorescently labelled 256 bp oligonucleotide substrates containing a site specific synthetic AP site (tetrahydrofuran, THF) in the 601 DNA sequence by molecular cloning (Figure 1A), similar to that previously described (25). These two substrates when bound with a histone octamer would generate mononucleosome substrates containing the synthetic AP site where the DNA backbone is either inwardly facing (THF-in), or outwardly facing (THF-out) from the histone core (Supplementary Figure S1).

**Figure 2.**
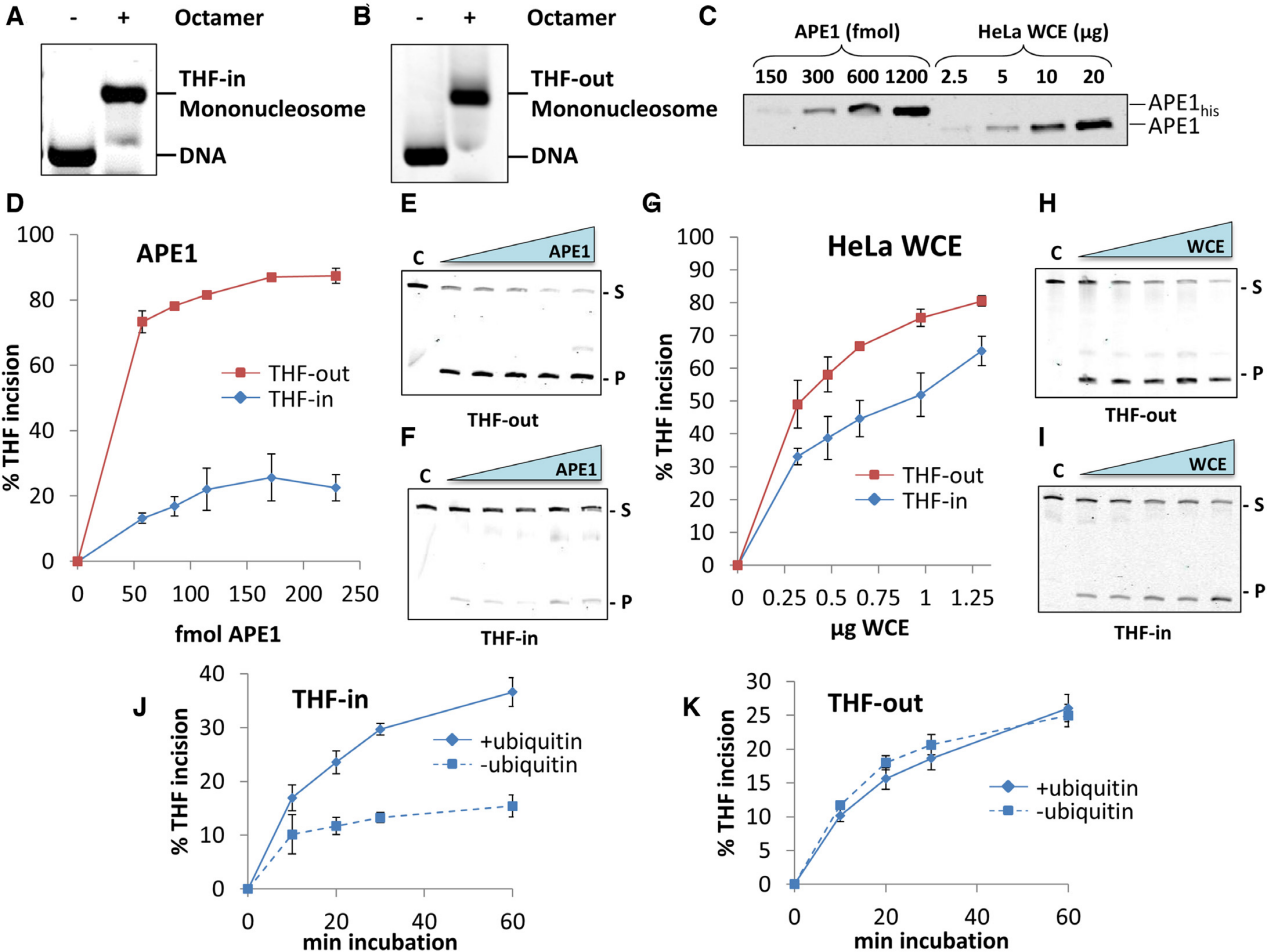
HeLa WCE more efficiently incises a THF-in mononucleosome substrate than recombinant APE1. (**A**, **B**) Mononucleosome DNA substrates containing THF with the backbone inwardly facing (THF-in) or outwardly facing (THF-out) were prepared by salt dialysis following incubation of the respective DNA substrates with histone octamer, and analysed by agarose gel electrophoresis. (**C**) Quantitative analysis of the levels of APE1 in HeLa WCE by comparison against increasing levels of recombinant APE1 by immunoblotting. Incision of THF-out and THF-in mononucleosome substrates (50 fmol) by increasing amounts of (**D**–**F**) recombinant APE1 and (**G**–**I**) HeLa WCEt. Shown is the mean percent substrate incision ± S.D. from at least three independent experiments, and also representative images from the respective gels. Time course of incision of (**J**) THF-in and (**K**) THF-out mononucleosome substrates (50 fmol) by HeLa whole cell extract (1.3 and 0.16 μg, respectively).

In the first instance, the free DNA substrates were analysed for their ability to be incised by both recombinant APE1, and APE1 present within HeLa WCE. We demonstrate that the free THF-out substrate is slightly more efficiently cleaved by APE1 (∼1.7-fold on average) than the free THF-in substrate (Figure 1B–D), indicating that there is a slight sequence bias in recognition of the AP site in the THF-out free DNA. The difference in cleavage of both substrates by APE1 present in HeLa WCE however, was less pronounced (Figure 1E–G). *Xenopus laevis* histone proteins (H2A, H2B, H3 and H4) were individually expressed in *Escherichia coli* and purified under denaturing conditions, refolded and histone octamer generated and purified using gel filtration chromatography (Supplementary Figure S2). The free DNA substrates were consequently bound with histone octamer to generate the respective mononucleosome substrates (Figure 2A and B), in which the AP site is 10 bp away from the nucleosome dyad. In order to compare the relative efficiency of APE1 and APE1 present within HeLa WCE to cleave these substrates, we quantified the levels of APE1 present in WCE by quantitative immunoblotting. We show that there is approximately 60 fmol APE1/μg HeLa WCE (Figure 2C). On examination of THF-in versus THF-out, we demonstrate that there is a clear difference in incision of these two mononucleosome substrates by recombinant APE1, in which the THF-out is significantly (4–5-fold) more efficiently cleaved than THF-in. Indeed incision of THF-in reaches a maximum of ∼25% using >120 fmol APE1 whereas ∼75% incision of THF-out is achieved using only 60 fmol APE1 (Figure 2D–F). This clearly demonstrates that incision of an AP site by APE1 with the DNA backbone facing inwards towards the histone core is significantly impeded. When using HeLa WCE, reactions were supplemented with factors to support a number of histone post-translational modifications, including phosphorylation (ATP), poly(ADP-ribosylation) (NAD) and ubiquitylation (E1 activating enzyme, nine E2 conjugating enzymes and ubiquitin), but which would also support ATP-dependent chromatin remodelling enzymes. We observed that the difference in cleavage of the two mononucleosome substrates (THF-out and THF-in) by HeLa WCE is still evident (∼1.4-fold on average), although this is significantly less that that observed with recombinant APE1 alone (∼4.2-fold on average). Indeed comparing 1 μg HeLa WCE generates cleavage of ∼75% and ∼50% of THF-out versus THF-in, respectively (Figure 2G–I). It is important to note, as mentioned above, that 1 μg HeLa WCE contains the equivalent amount of ∼60 fmol recombinant APE1 and that the same comparable level of incision of the THF-out mononucleosome substrate was observed when comparing these data points. In contrast, the incision of THF-in mononucleosome substrate by 1 μg HeLa WCE and 60 fmol recombinant APE1 is ∼4-fold different (∼50% versus 13%, respectively). Interestingly we discovered that effective incision of the THF-in mononucleosome substrate by HeLa WCE is significantly dependent on supplementing reactions with ubiquitin (Figure 2J, Supplementary Figure S3A and B), and further enhanced by addition of E1 and E2 enzymes that promote protein ubiquitylation (data not shown). However, these factors had no impact on the rate of incision of the THF-out mononucleosome substrate (Figure 2K, Supplementary Figure S3C and D). Nevertheless, these findings support our hypothesis that incision of an AP site within a mononucleosome substrate that is inaccessible to recombinant APE1 (THF-in), is more efficiently incised by APE1 in WCE due to the presence of histone modifiers and/or chromatin remodelling factors, largely in a ubiquitylation-dependent manner.

### Purification of factors promoting AP site accessibility

**Figure 3.**
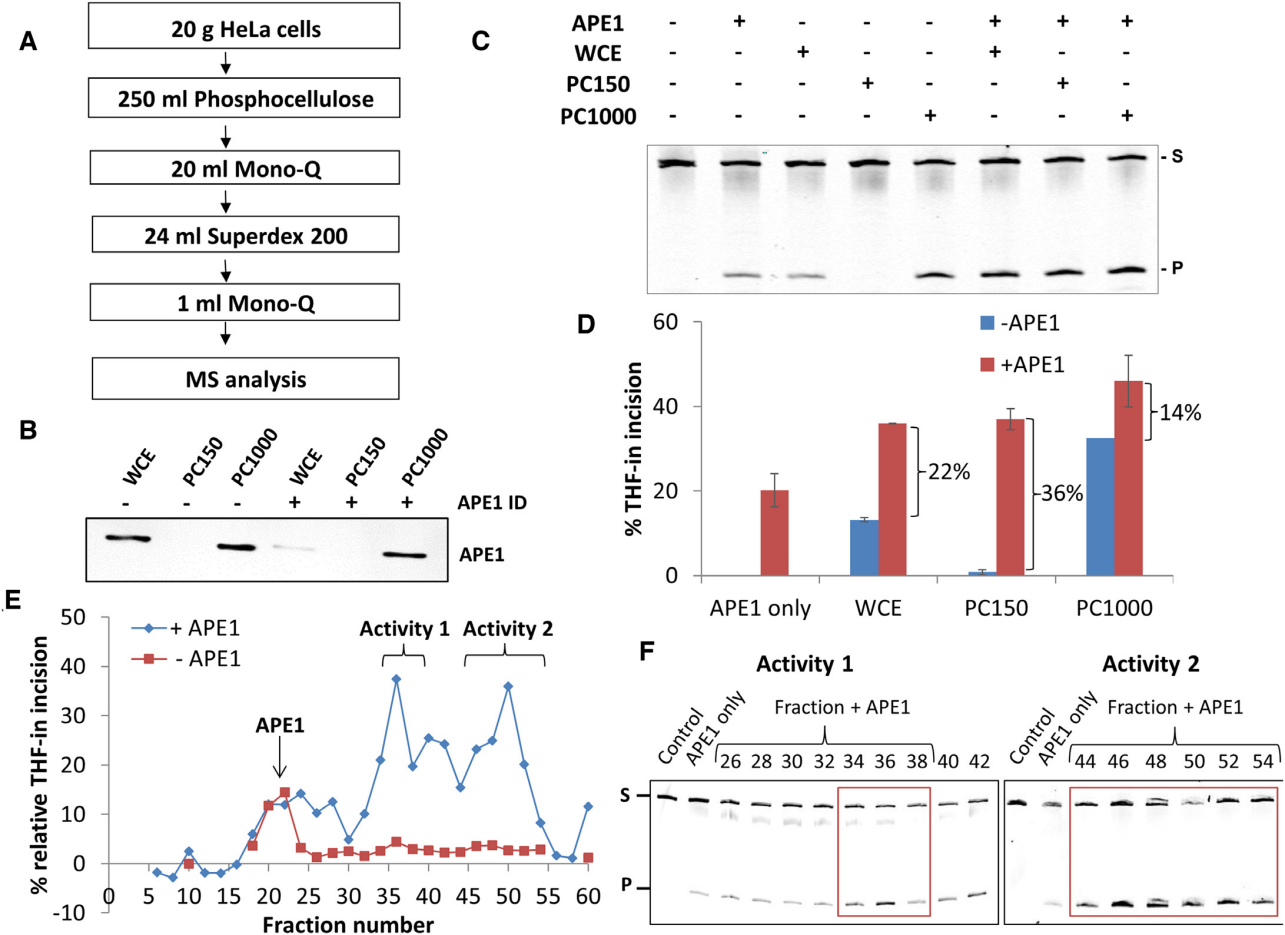
Stimulatory activities are present in HeLa WCE that enhance incision of THF-in mononucleosome substrate by recombinant APE1. (**A**) Purification scheme for the isolation of stimulatory activities from HeLa WCE using column chromatography, finishing with mass spectrometry (MS) analysis of active protein fractions. (**B**) Immunoblotting analysis of APE1 within WCE and fractions (10 μg) generated by low salt (PC150) and high salt (PC1000) elution of proteins from Phosphocellulose chromatography. Fractions were also immunodepleted using APE1-specific antibodies (APE1 ID), although this was only partially effective using the PC1000 fraction. (**C, D**) Incision of the THF-in mononucleosome substrate (50 fmol) by WCE, PC150 and PC1000 (0.16 μg) in the absence and presence of recombinant APE1 (60 fmol). (**D**) Shown is the mean percent substrate incision from two independent experiments, along with the difference in THF incision by the extracts in the absence and presence of recombinant APE1. (**E, F**) Stimulation of APE1-dependent incision of the THF-in mononucleosome substrate by fractionated proteins generated from Mono-Q chromatography. (**E**) Shown is the mean percent substrate incision from two independent experiments in the absence (red line) or presence (blue line) of recombinant APE1, normalized to 0 after subtraction of the incision observed with recombinant APE1 only (∼20%). (**F**) A representative image from the respective gels is shown highlighting the majority of the stimulatory activities present in fractions 34–38 and 44–54. The respective control reactions performed in the absence of any fraction (Control) and with APE1 only are indicated.

Given our evidence that factors are present within WCE that are capable of stimulating APE1 activity on the THF-in mononucleosome substrate, we utilized an unbiased approach using a purification scheme involving separation of proteins in WCE by different ion-exchange and size exclusion chromatography columns (Figure 3A) to purify and identify these stimulatory factors. Protein fractions were then examined for their ability to stimulate incision of the THF-in mononucleosome substrate by recombinant APE1. Since WCE contains endogenous APE1, we monitored for the presence of APE1 by immunoblotting and also activity of the purified fractions alone at several stages during the purification procedure, to ensure that stimulatory factors were being isolated rather than endogenous APE1 protein. From the first chromatography stage through separation of HeLa WCE via a Phosphocellulose column, the majority of APE1 protein present in WCE eluted within the high salt elution fraction (PC1000) in comparison to the low salt elution fraction (PC150), consistent with its high DNA binding affinity (Figure 3B). We attempted to immunodeplete APE1 to minimise the background level of endonuclease activity in the fractions but this was only successful from HeLa WCE, and not from PC1000 (Figure 3B). However, the presence of APE1 within the respective fractions was consistent with the degree of activity of these alone against the THF-in mononucleosome substrate, in which only WCE and the PC1000 fraction demonstrated significant incision activity (Figure 3C, compare lanes 3–5) and where the activity of PC1000 was greatest due to the increased amount of APE1 (Figure 3D, compare blue bars). The baseline activity level of these fractions was then compared in the presence of recombinant APE1, which alone generated ∼20% incision of the THF-in mononucleosome substrate (Figure 3C, lane 2; Figure 3D, first red bar). We discovered that substrate incision was increased by 14–22% using WCE and PC1000 fractions in the presence of recombinant APE1, which can be attributed to the additional APE1 protein (Figure 3C, compare lanes 6–8; Figure 3D, compare blue versus red bars). However, incision activity was stimulated by ∼36% using the PC150 fraction, demonstrating that factors improving the accessibility of the THF-in mononucleosome substrate to recombinant APE1 were largely present in this fraction. In contrast, performing the same experiment using the THF-in free DNA substrate revealed that the increase in incision between the absence and presence of recombinant APE1 was ∼20% using the WCE, PC150 or PC1000 fractions (Supplementary Figure S4A and B). This is consistent with the incision activity observed with APE1 only, and demonstrates that factors stimulating APE1 within PC150 are specifically enhancing its incision towards the THF-in mononucleosome substrate.

**Figure 4.**
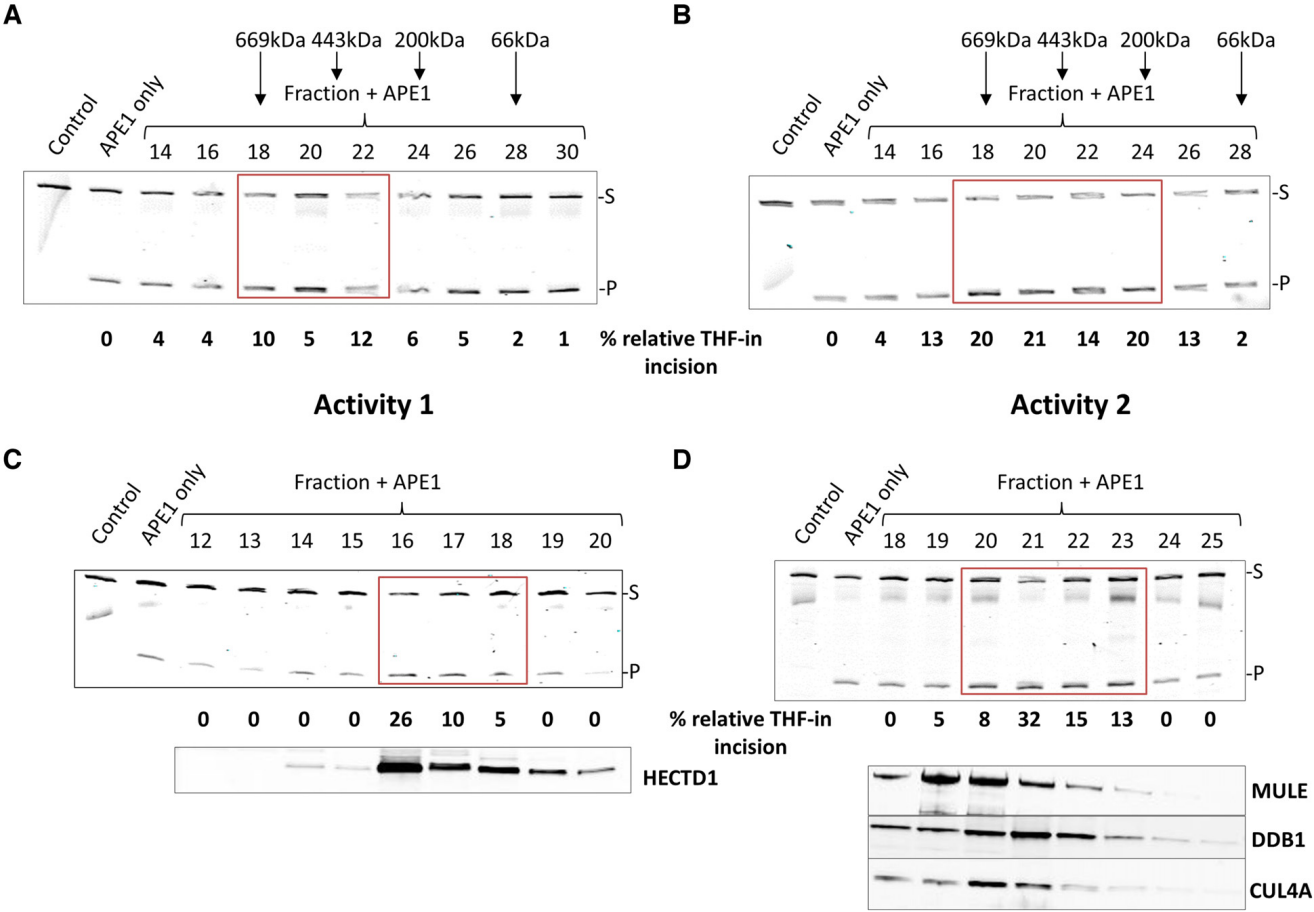
Purification and identification of HECTD1 and Cul4A-DDB1 complex as potential stimulators of incision of THF-in mononucleosome substrate by recombinant APE1. (**A–D**) Stimulation of APE1-dependent (60 fmol) incision of the THF-in mononucleosome substrate (50 fmol) by fractionated proteins generated from Activity 1 or Activity 2 by sequential (**A** and **B**) Superdex 200 and (**C** and **D**) Mono-Q chromatography, respectively. (**A, B**) Positions of elution of known molecular weight protein standards are indicated above the fractions generated by gel filtration chromatography. Shown below each figure is the mean percent THF-in substrate incision from two independent experiments, normalized to 0 after subtraction of the incision observed with recombinant APE1 only (∼20%). Fractions from the final Mono-Q chromatography stage were also analysed by immunoblotting for the presence of (**C**) HECTD1 and (**D**) Mule, Cul4A and DDB1 and are aligned with the THF-in mononucleosome activity profiles.

The PC150 fraction was subsequently separated by ion exchange (Mono-Q) chromatography that yielded ∼80 protein fractions (Supplementary Figure S5A), which were examined in the presence of recombinant APE1 for stimulation of activity against the THF-in mononucleosome substrate. This analysis revealed the presence of three major stimulatory activities (Figure 3E, blue line) that generated substrate incision that was above the level observed using recombinant APE1 only (20%; subtracted from the fraction data to yield relative THF-in incision, indicative of chromatin remodelling activity). However, on analysis of the fractions in the absence of recombinant APE1, a peak of incision activity was observed in fractions 16–22 (Supplementary Figure S5B and Figure 3E, red line), which was attributable to the presence of residual endogenous APE1 as confirmed by immunoblotting for APE1 (Supplementary Figure S5C). We therefore focussed on the two remaining stimulatory activities (designated Activity 1 and Activity 2) which were present in fractions ∼34–38 and ∼44–54 (Figure 3F), respectively for further purification. Following size exclusion (Superdex 200) chromatography of both activities (Supplementary Figure S6A and B), a relative increase of ∼10–20% incision of the THF-in mononucleosome substrate by recombinant APE1 suggestive of chromatin remodelling activity was observed using fractions 18–22 (Activity 1; Figure 4A) and 18–24 (Activity 2; Figure 4B). This purification stage revealed that the stimulatory activities were equivalent to proteins of ∼400–600 kDa in molecular weight. These activities were still retained following separation of protein fractions on a second ion exchange (Mono-Q) chromatography column (Supplementary Figure S6C-D), as shown in fractions 16–18 (Activity 1; Figure 4C) and 20–23 (Activity 2; Figure 4D) where a relative ∼26–32% increase in THF-in incision was observed. Following this final chromatography stage, highly purified protein fractions were subsequently analysed by mass spectrometry in order to identify potential candidate proteins. Given that we originally identified that accessibility to the THF-in mononucleosome substrate was largely dependent on factors supporting ubiquitylation (Figure 2J, Supplementary Figure S3A and B), our particular focus was on the identification of E3 ubiquitin ligases present in the fractions as potential chromatin remodelling enzymes. Indeed, in the list of proteins present in fractions from Activity 1 were the E3 ubiquitin ligases HECT domain containing E3 protein ligase 1 (HECTD1) and C-terminus of Hsp70-interacting protein (CHIP; Table 1), whereas in Activity 2 Mcl-1 ubiquitin ligase E3 (Mule) and DNA damage-binding protein 1 (DDB1; a component of the Cul4 E3 ligases; Table 2) were identified. We were subsequently able to show by immunoblotting that the presence of HECTD1 aligns well with stimulatory activity observed in the final Mono-Q fractions purified from Activity 1 (Figure 4C, lower panel), and that Cul4A and DDB1 (but not Mule) aligns well with Activity 2 purified fractions (Figure 4D, lower panel). Given the fact that Cul4A-DDB1 forms a scaffold for a large number (>90) E3 ubiquitin ligases, and in the absence of an identified WD-40 repeat protein that provides E3 ubiquitin ligase specificity, we focussed our attention on HECTD1 as a possible novel factor involved in stimulating BER activity both *in vitro* and *in vivo*.

**Table 1.** Mass spectrometry analysis of selected proteins within Activity 1

**Accession**	**Description**	**Mascot Score**
P07900	Heat shock protein HSP90-alpha	3426
P08238	Heat shock protein HSP90-alpha	3379
P49736	DNA replication licensing factor MCM2	652
P33993	DNA replication licensing factor MCM7	645
**Q9ULT8**	**E3 ubiquitin protein ligase HECTD1**	**591**
**Q9UNE7**	**E3 ubiquitin protein ligase CHIP**	**536**

**Table 2.** Mass spectrometry analysis of selected proteins within Activity 2

**Accession**	**Description**	**Mascot score**
O15355	Protein phosphatase 1G	1581
Q7KZ85	Transcription elongation factor SPT6	1446
**P49792**	**E3 ubiquitin protein ligase MULE**	**1041**
Q86VP6	Cullin-associated NEDD8-dissociated protein 1	241
**Q16531**	**DNA damage binding protein 1**	**167**

### Identification of HECTD1 as an enzyme promoting AP site accessibility

**Figure 5.**
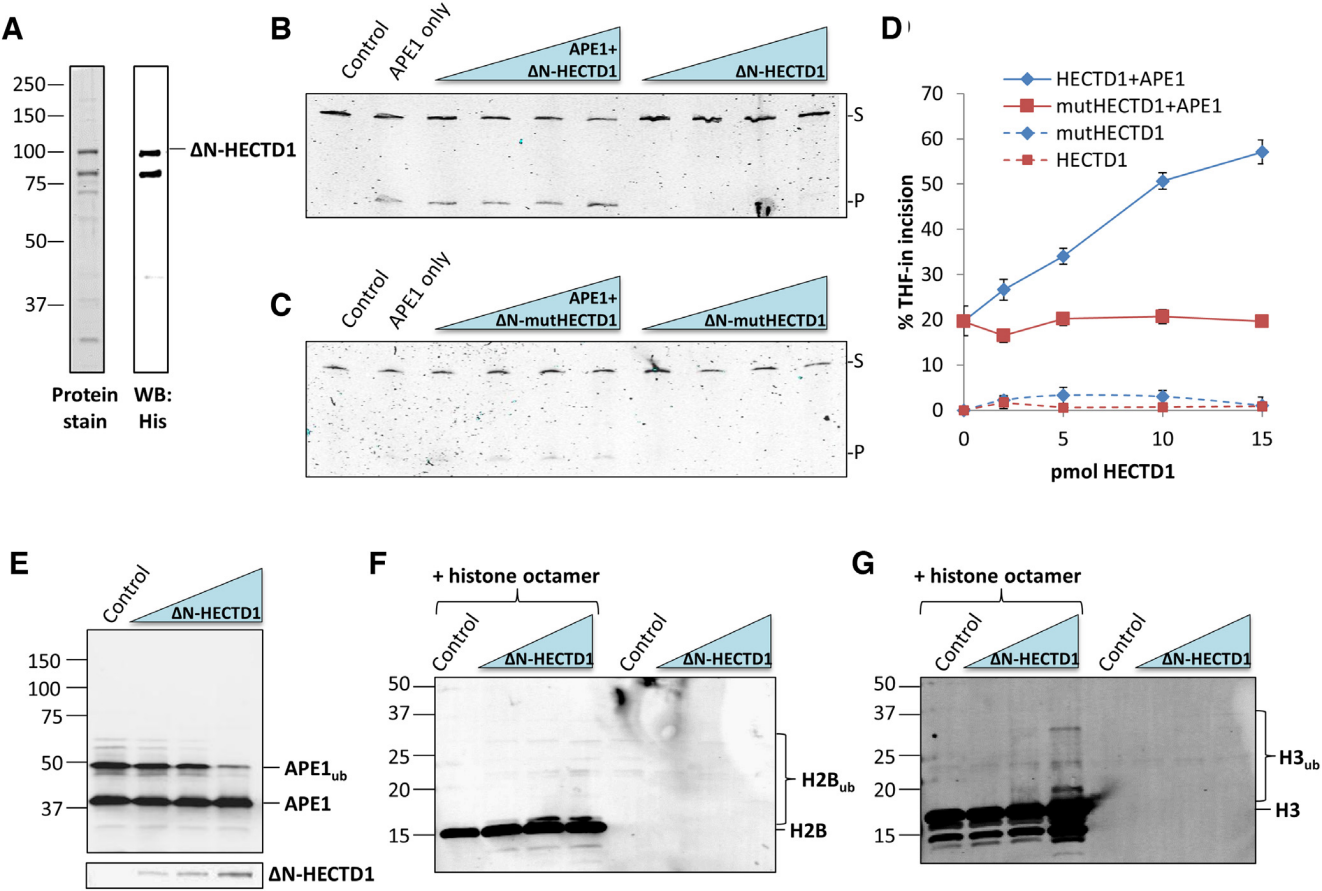
HECTD1 promotes incision of THF-in mononucleosome substrate by recombinant APE1 *in vitro*. (**A**) A C-terminal truncation of murine HECTD1 (amino acids 1762–2610; ΔN-HECTD1) was overexpressed in bacterial cells and purified using His-tag chromatography. Shown is the total protein as revealed by Instant Blue staining (left panel), as well as detection of the protein by immunoblotting using histag antibodies (right panel). (**B–D**) Stimulation of APE1-dependent (60 fmol) incision of the THF-in mononucleosome substrate (50 fmol) by increasing amounts of (**B**) wild type ΔN-HECTD1 and (**C**) catalytically inactive E3 ligase mutant ΔN-HECTD1 (C2579G; ΔN-mutHECTD1). (**D**) Shown is the mean percent substrate incision ± S.D. from three independent experiments by ΔN-HECTD1/ΔN-mutHECTD1 in the absence and presence of APE1. The respective control reactions were performed in the absence of any protein (Control) and with recombinant APE1 only. (**E**) *In vitro* ubiquitylation assays containing recombinant APE1 (5.9 pmol) in the presence of increasing amounts of ΔN-HECTD1 (2.8–14.1 pmol). Samples were separated by SDS-PAGE and analysed by immunoblotting using APE1 antibodies, or with antibodies targeting HECTD1 (lower panel). (**F, G**) *In vitro* ubiquitylation assays containing histone octamer (2 pmol) in the presence of increasing amounts of ΔN-HECTD1 (2.8–14.1 pmol). Samples were separated by SDS-PAGE and analysed by immunoblotting using (**F**) histone H2B or (**G**) histone H3 antibodies. The respective control reactions (Control) were performed in the absence of any HECD1.

We firstly attempted to immunodeplete HECTD1 from Mono-Q fractions containing the peak stimulatory activity for APE1 (Figure 4C, fraction 16), however we were unable to optimize this although a small depletion of the protein did lead to a corresponding small decrease in stimulation of incision of the THF-in mononucleosome substrate by recombinant APE1 (Supplementary Figure S7A–C). We next focussed on purifying recombinant HECTD1 for utilization in the *in vitro* BER assays. Using a mammalian expression plasmid expressing murine HECTD1 (30), we recloned the C-terminal cDNA sequence containing the E3 ubiquitin ligase HECT domain (amino acids 1762–2610; Supplementary Figure S8A) into a bacterial expression plasmid. The truncated protein (ΔN-HECTD1; ∼96 kDa) was overexpressed and purified from bacterial cells but was found to undergo partial degradation to a protein of a slightly small molecular weight (∼85 kDa; Figure 5A and Supplementary Figure S8B and C). Nevertheless, we determined that ΔN-HECTD1 is able to significantly stimulate the activity of recombinant APE1 against the THF-in mononucleosome substrate from 20 to 57% (Figure 5B, lanes 3–6 and Figure 5D). In contrast, there was no impact of ΔN-HECTD1 alone on incision of the THF substrate (Figure 5B, lanes 7–10 and Figure 5D). We also generated and purified an inactive E3 ligase mutant (C2579G; previously reported (30)) of ΔN-HECTD1 (ΔN-mutHECTD1; Supplementary Figure S8D and E). We demonstrate that the bacterially expressed and purified mutant protein, in comparison to the wild type protein, is unable to stimulate APE1 incision of the THF-in mononucleosome substrate (Figure 5C, lanes 3–6 and Figure 5D). This provides evidence that the E3 ubiquitin ligase activity of HECTD1 is required for promoting THF-in incision within mononucleosomes by APE1. Additionally, we have also acquired evidence that HECTD1 is able to stimulate the excision of an inwardly facing thymine glycol (TG-in) containing mononucleosome substrate from 24 to 60% by the DNA glycosylase endonuclease III homologue, NTH1 (Supplementary Figure S9A–C), suggesting that HECTD1 may be able to promote multiple stages of BER.

To establish that HECTD1 is specifically targeting the histones for ubiquitylation, we first checked that APE1 itself was not being ubiquitylated which is potentially increasing AP endonuclease enzymatic activity. Whilst we observed E2-dependent monoubiquitylation of APE1 in the absence of HECTD1 *in vitro* (Figure 5E, lane 1), which we have observed previously (31), an increasing presence of ΔN-HECTD1 did not stimulate APE1 ubiquitylation and in fact supressed E2-dependent monoubiquitylation at the highest protein concentration (Figure 5E, lanes 2–4). We then examined *in vitro* ubiquitylation of histone proteins within the octamer, and reveal evidence that ΔN-HECTD1 appeared to cause a modest increase in mono/di-ubiquitylation of histone H2B (Figure 5F, lanes 2–4) but more convincingly promotes histone H3 polyubiquitylation (Figure 5G, lanes 2–4). No significant cross-reactivity of the respective antibodies was observed in reactions in the absence of the histone octamer (Figure 5F and G, lanes 5–8). Furthermore, we demonstrated that histones H2A or H4 do not seem to be a target for ubiquitylation by HECTD1 (Supplementary Figure 10A and B), although the poor quality of the antibodies used does not allow us to fully draw this conclusion. However, with the availability of the current data, this suggests that HECTD1 is promoting accessibility of APE1 to the THF-in mononucleosome substrate through histone H2B/H3 ubiquitylation.

### HECTD1 is required for efficient repair of DNA base damage and SSBs

**Figure 6.**
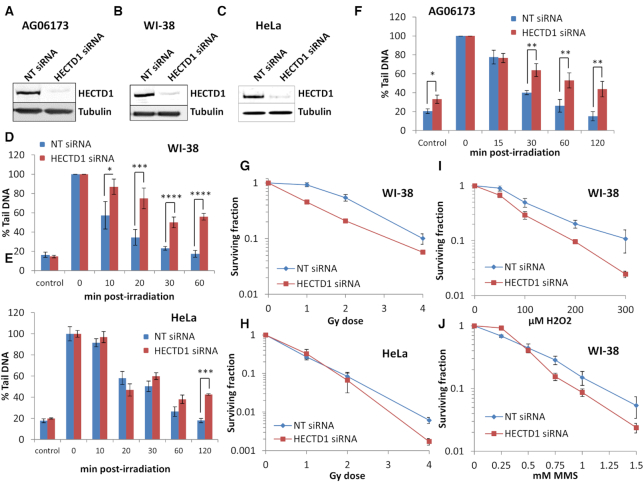
HECTD1 controls the efficiency of DNA damage repair in response to ionizing radiation. (**A**) AG06173, (**B**) WI-38 or (**C**) HeLa cells were treated with 40 nM non-targeting control siRNA (NT) or HECTD1 siRNA for 48 h and extracts analysed by immunoblotting with HECTD1 or tubulin antibodies as a loading control. (**D**) WI-38, (**E**) HeLa or (**F**) AG06173 cells were treated with 40 nM NT control or HECTD1 siRNA for 48 h, irradiated with 1.5 Gy x-rays and the levels of DNA single strand breaks and alkali-labile sites measured at various time points post-irradiation by the alkaline comet assay. Shown is the % tail DNA±S.D. normalized to the levels seen immediately post-IR which was set to 100% from three independent experiments. *P < 0.05, **P < 0.02, ***P < 0.005, ^****^P < 0.001 as analysed by a two sample *t*-test. Clonogenic survival of (**G**) WI-38 or (**H**) HeLa cells after NT siRNA or HECTD1 siRNA following x-ray irradiation. Clonogenic survival of WI-38 cells after NT siRNA or HECTD1 siRNA following (**I**) H_2_O_2_ or (**J**) MMS. Shown is the mean surviving fraction ± S.E. from at least three independent experiments.

To examine the requirement for HECTD1 in promoting BER in cells, we used siRNA to effectively deplete the cellular levels of the protein in AG06173 and WI-38 normal lung fibroblasts, and also in HeLa cervical carcinoma cells versus a non-targeting (NT) control siRNA (Figure 6A–C). Following treatment of cells with x-ray irradiation and examining the kinetics of DNA repair by the alkaline comet assay, we show that depletion of HECTD1 caused a significant delay in the repair of SSBs and alkali-labile sites versus NT siRNA treated WI-38 and AG06173 cells. This is evidenced by statistically significant higher levels of DNA damage in WI-38 cells at 10–60 minutes post-irradiation (Figure 6D) and in AG06173 at 30–120 min post-irradiation (Figure 6F). Repair of SSBs and alkali-labile sites in HECTD1 depleted versus NT siRNA treated HeLa cells was also delayed, although this was only statistically significant at 120 min post-irradiation (Figure 6E). An absence of HECTD1, versus NT control siRNA, also caused an elevation in the levels of SSBs and alkali-labile sites in control, unirradiated AG06173 cells (Figure 6F) suggesting a specific requirement for HECTD1 in the efficient processing of endogenous DNA damage in these cells. We then measured radiosensitivity of cells using clonogenic survival assays, although were unable to achieve this in AG06173 cells as these do not form defined colonies. However, we were able to show significantly decreased survival of HECTD1 depleted WI-38 cells compared to NT siRNA treated cells in response to x-ray irradiation (Figure 6G and Supplementary Figure S11A). In contrast, HeLa cells only showed a marked increase in radiosensitivity of HECTD1 depleted cells versus NT siRNA treated cells at a high dose (4 Gy) of x-rays (Figure 6H and Supplementary Figure S11B). This difference in radiosensitivity of WI-38 versus HeLa cells in the absence of HECTD1, appears to be reflected in the comet assay data where there is a much greater dependency on HECTD1 for efficient processing of SSBs and alkali-labile sites in normal lung fibroblasts (Figure 6D-F). Additionally, HECTD1 depleted WI-38 cells are also more sensitive to the cell killing effects of hydrogen peroxide and methylmethanesulfonate (MMS; Figure 6I and J, Supplementary Figure 12A and B) that generate oxidative DNA base damage and DNA base alkylation that is processed through BER. In summary, our data now demonstrates the importance of HECTD1 in the efficient processing of DNA base damage and SSBs through the BER pathway which is required for maintaining cell survival in response to exogenous DNA damaging agents.

## DISCUSSION

Our cellular DNA is subject to constant attack by ROS generated through cellular oxidative metabolism and by exogenous sources such as ionizing radiation. BER plays a vital role in repairing DNA base damage and SSBs, thus reducing the frequency of cellular mutations and ultimately in preventing development of diseases, including cancer. The major enzymes directly involved in BER are now well established, although how the BER process is co-ordinated within chromatin where the DNA is packaged with histone proteins is not entirely understood. Accumulating biochemical evidence has demonstrated that DNA base damage, particularly with the DNA backbone facing inwards towards the histone octamer, is less efficiently repaired by recombinant BER proteins *in vitro* (reviewed in (32)). This suggests that chromatin remodelling is required for efficient DNA base damage processing within chromatin, particularly in occluded regions of the DNA. Indeed, this is supported by evidence suggesting that the chromatin remodelling enzymes SWI/SNF and RSC can increase the activities of BER proteins on mononucleosome substrates *in vitro* (19–21), although there is no significant evidence to date to suggest that these enzymes function during BER *in vivo*.

In this manuscript, we demonstrate that a THF-in mononucleosome substrate that is inefficiently incised by recombinant APE1, is processed more effectively by APE1 present within HeLa WCE particularly in the presence of factors supporting ubiquitylation (E1 and E2 enzymes, plus ubiquitin). Using an unbiased screen utilizing fractionated HeLa WCE, we subsequently purified the E3 ubiquitin ligase HECTD1 as one of the major enzymes enhancing incision of the THF-in mononucleosome substrate by APE1 *in vitro*. This was confirmed using purified recombinant HECTD1 protein but not by an E3 ligase inactive mutant, demonstrating the requirement for ubiquitylation in promoting THF-in incision and we provide evidence that this is potentially mediated via histone H2B/H3 ubiquitylation. We also demonstrated that HECTD1 stimulates NTH1-dependent excision of a TG-in mononucleosome substrate. Furthermore, we show that HECTD1 is essential for efficient repair of DNA base damage and SSBs, and in promoting cell survival particularly in normal lung fibroblasts following x-ray irradiation, H_2_O_2_ and MMS. Thereby, we reveal HECTD1 as a new player in the BER process within chromatin, where it acts to enhance repair of DNA damage within occluded DNA regions.

HECTD1 is a 289 kDa protein and a member of the HECT domain containing E3 ubiquitin ligases. HECTD1 was first characterized to target HSP90 for ubiquitylation and control the cellular localization and secretion of the protein necessary for regulation of the behaviour of cranial mesenchyme cells (30). Following this study, HECTD1 was reported to catalyse polyubiquitylation of the adenomatous polyposis coli (APC) protein in HEK293 cells which was required for interaction with Axin, and that HECTD1 functions as a negative regulator of Wnt/β-catenin signalling (33). This was supported by the observed downregulation of Wnt pathway activity in LN-229 and LN-428 glioblastoma cells overexpressing HECTD1, but also that the deubiquitylation enzyme USP15 was directly involved in stabilization of HECTD1 (34). More recently, HECTD1 has been demonstrated to negatively impact on endothelial–mesenchymal transition in response to silicon dioxide in MML1 mouse lung cells and to inhibit cell proliferation and migration (35). Furthermore, HECTD1 has been shown to promote ubiquitylation-dependent degradation of ACF7 and that HECTD1 depletion in T47D breast cancer cells leads to increased ACF7 protein levels, enhanced epithelial–mesenchymal transition and promotes tumour growth, survival and metastasis (36). Interestingly, the latter study demonstrated a correlation of low HECTD1 protein expression and shorter disease-free survival in breast cancer patients, and low mRNA levels of *hectd1* with reduced survival in multiple cancer types, including breast, lung and brain. The observations in our study that HECTD1 is required to promote efficient repair of DNA base damage, would also suggest that low HECTD1 levels could contribute to increased mutagenesis and carcinogenesis, and increase the likelihood of metastasis. Therefore we now highlight an essential role for HECTD1 in the cellular DNA damage response, which in addition to reports demonstrating roles in controlling cell signalling, proliferation and migration, cumulatively support the vital function that HECTD1 plays in normal cell physiology.

There are a few unanswered questions we have yet to fully address regarding the role of HECTD1 in promoting BER *in vivo*. The first is the precise target for histone ubiquitylation since our preliminary evidence, at least *in vitro*, would suggest that HECTD1 can promote histone H2B mono/di-ubiquitylation and to a greater extent histone H3 polyubiquitylation. We have recently screened for changes in a number of histone post-translational modifications in response to radiation of different ionization densities, but also following hydrogen peroxide (37). Whilst this analysis was limited to antibodies targeting site-specific modifications, generally we did not observe any dramatic changes in the kinetics of these histone modifications (which included histone H2B lysine 120, but also histones H2A and H2AX lysine 119 monoubiquitylation) in response to γ-irradiation or oxidative stress. In fact acetylation and di/tri-methylation of histone H3 were also relatively unchanged under these conditions, suggesting that if histone H3 is a target for ubiquitylation by HECTD1 in response to DNA damage, then there is no current evidence of any cross-talk between these specific modifications. Therefore, our major immediate focus will be to identify and characterize the site-specific histone modification targeted by HECTD1. A second question is how HECTD1 is able to trigger histone ubiquitylation in response to changes in DNA damage induced by endogenous and exogenous agents, and whether this mechanism is limited to just DNA base damage and SSBs. Given the evidence described above, this sensing mechanism may potentially involve regulation by USP15 although this enzyme appears to be controlling stabilization of HECTD1 rather than modulating its activity towards specific targets. Therefore, we need to understand whether HECTD1 is directed to sites of DNA damage, or whether the protein is actively scanning along chromatin searching for changes or distortions in the DNA. Thirdly, and related to that described above, the specificity of HECTD1 in promoting the repair of DNA damage particularly through the BER pathway needs to be addressed. Our data demonstrating that HECTD1 is able to promote both NTH1 and APE1 during DNA base excision and strand incision, respectively *in vitro* as well as the survival of WI-38 cells in response to x-ray irradiation, H_2_O_2_ and MMS, would suggest that HECTD1 plays a general role during BER. It would also suggest that HECTD1 is not recruited to DNA damage sites through interaction with a specific BER protein, but rather through its association with the chromatin and DNA damage itself. However, the specificity of HECTD1 in stimulating BER needs to be confirmed using multiple different DNA lesion-containing mononucleosome substrates (e.g. DNA base damage, single nucleotide gap and SSB) in the out and inwardly facing orientations relative to the histone core and the appropriate BER enzymes *in vitro*. Whether HECTD1 is responsive to other types of DNA damage, such as bulky DNA lesions processed by nucleotide excision repair or DNA double strand breaks, also needs examining. Nevertheless, these questions are the focus of our current and ongoing investigations.

Our purification strategy utilizing HeLa WCE also suggested that a Cul4A-DDB1 containing complex may play a role in promoting repair of DNA base damage within mononucleosomes. It is well known that these proteins along with Roc1 form a platform for a large number of E3 ubiquitin ligase complexes. In fact, the specificity of these enzymes for target substrates is defined by a WD-40 repeat protein and that >90 such complexes exist (38,39). Indeed, we recently demonstrated that serine–threonine kinase receptor associated protein (STRAP) is an example of one such protein that promotes ubiquitylation-dependent degradation of polynucleotide kinase phosphatase and regulates oxidative DNA damage repair (40). Given the large number of Cul4A-DDB1 E3 ligase complexes and their multiple cellular roles making it difficult to interpret cellular studies by targeting specifically Cul4A or DDB1, and in the absence of an identified WD-40 repeat protein in our purified protein fractions, we have yet to further investigate this observation. However, Cul4A–DDB1–Roc1 has previously been demonstrated to ubiquitylate histone H3 and H4 in response to UV irradiation, where it is required for nucleosome remodelling to enhance the repair of thymine dimers (41). Therefore, we aim to further interrogate these purified active fractions, through either additional protein purification steps or via immunoprecipitation of the Cul4A–DDB1 complex, in order to identify by mass spectrometry the WD-40 repeat protein which is specifically associated with the core components. The impact of depletion of this specific protein would then allow us to examine its specific role in the cellular DNA damage response.

In summary, we have identified a crucial role for HECTD1 in enhancing the repair of DNA base damage and SSBs within occluded sites in chromatin. This has revealed further evidence that BER can be stimulated via histone post-translational modifications and chromatin remodelling, specifically promoted by histone H2B/H3 ubiquitylation by HECTD1, and which is required for an efficient cellular DNA damage response and in promoting cell survival in response to genotoxic stress.

## Supplementary Material

gkz1129_Supplemental_File

## Notes

Present address: Laura Bennett, North West Cancer Research Institute, Bangor University, Brambell Building, Deiniol Road, Bangor LL57 2UW, UK.

## References

[1] Lindahl T. Instability and decay of the primary structure of DNA. Nature. 1993; 362:709–715. 8469282 10.1038/362709a0

[2] Parsons J.L., Dianov G.L. Co-ordination of base excision repair and genome stability. DNA Repair (Amst.). 2013; 12:326–333. 23473643 10.1016/j.dnarep.2013.02.001

[3] Carter R.J., Parsons J.L. Base excision repair, a pathway regulated by posttranslational modifications. Mol. Cell Biol. 2016; 36:1426–1437. 26976642 10.1128/MCB.00030-16 PMC4859697

[4] Horton J.K., Watson M., Stefanick D.F., Shaughnessy D.T., Taylor J.A., Wilson S.H. XRCC1 and DNA polymerase beta in cellular protection against cytotoxic DNA single-strand breaks. Cell Res. 2008; 18:48–63. 18166976 10.1038/cr.2008.7 PMC2366203

[5] Fung H., Demple B. A vital role for Ape1/Ref1 protein in repairing spontaneous DNA damage in human cells. Mol. Cell. 2005; 17:463–470. 15694346 10.1016/j.molcel.2004.12.029

[6] Rasouli-Nia A., Karimi-Busheri F., Weinfeld M. Stable down-regulation of human polynucleotide kinase enhances spontaneous mutation frequency and sensitizes cells to genotoxic agents. Proc. Natl. Acad. Sci. U.S.A. 2004; 101:6905–6910. 15100409 10.1073/pnas.0400099101 PMC406440

[7] Brem R., Hall J. XRCC1 is required for DNA single-strand break repair in human cells. Nucleic Acids Res. 2005; 33:2512–2520. 15867196 10.1093/nar/gki543 PMC1088068

[8] Clapier C.R., Cairns B.R. The biology of chromatin remodeling complexes. Annu. Rev. Biochem. 2009; 78:273–304. 19355820 10.1146/annurev.biochem.77.062706.153223

[9] Dianov G.L., Meisenberg C., Parsons J.L. Regulation of DNA repair by ubiquitylation. Biochemistry (Mosc.). 2011; 76:69–79. 21568841 10.1134/s0006297911010093

[10] Jackson S.P., Durocher D. Regulation of DNA damage responses by ubiquitin and SUMO. Mol. Cell. 2013; 49:795–807. 23416108 10.1016/j.molcel.2013.01.017

[11] Ahel D., Horejsi Z., Wiechens N., Polo S.E., Garcia-Wilson E., Ahel I., Flynn H., Skehel M., West S.C., Jackson S.P. et al. Poly(ADP-ribose)-dependent regulation of DNA repair by the chromatin remodeling enzyme ALC1. Science. 2009; 325:1240–1243. 19661379 10.1126/science.1177321 PMC3443743

[12] Polo S.E., Kaidi A., Baskcomb L., Galanty Y., Jackson S.P. Regulation of DNA-damage responses and cell-cycle progression by the chromatin remodelling factor CHD4. EMBO J. 2010; 29:3130–3139. 20693977 10.1038/emboj.2010.188 PMC2944064

[13] Xu Y., Price B.D. Chromatin dynamics and the repair of DNA double strand breaks. Cell Cycle. 2011; 10:261–267. 21212734 10.4161/cc.10.2.14543 PMC3048797

[14] Prasad A., Wallace S.S., Pederson D.S. Initiation of base excision repair of oxidative lesions in nucleosomes by the human, bifunctional DNA glycosylase NTH1. Mol. Cell Biol. 2007; 27:8442–8453. 17923696 10.1128/MCB.00791-07 PMC2169407

[15] Cole H.A., Tabor-Godwin J.M., Hayes J.J. Uracil DNA glycosylase activity on nucleosomal DNA depends on rotational orientation of targets. J. Biol. Chem. 2010; 285:2876–2885. 19933279 10.1074/jbc.M109.073544 PMC2807341

[16] Hinz J.M. Impact of abasic site orientation within nucleosomes on human APE1 endonuclease activity. Mutat. Res. 2014; 766–767:19–24. 10.1016/j.mrfmmm.2014.05.008 PMC4112536 25847267

[17] Rodriguez Y., Smerdon M.J. The structural location of DNA lesions in nucleosome core particles determines accessibility by base excision repair enzymes. J. Biol. Chem. 2013; 288:13863–13875. 23543741 10.1074/jbc.M112.441444 PMC3650422

[18] Odell I.D., Barbour J.E., Murphy D.L., Della-Maria J.A., Sweasy J.B., Tomkinson A.E., Wallace S.S., Pederson D.S. Nucleosome disruption by DNA ligase III-XRCC1 promotes efficient base excision repair. Mol. Cell Biol. 2011; 31:4623–4632. 21930793 10.1128/MCB.05715-11 PMC3209256

[19] Menoni H., Gasparutto D., Hamiche A., Cadet J., Dimitrov S., Bouvet P., Angelov D. ATP-dependent chromatin remodeling is required for base excision repair in conventional but not in variant H2A.Bbd nucleosomes. Mol. Cell Biol. 2007; 27:5949–5956. 17591702 10.1128/MCB.00376-07 PMC1952146

[20] Menoni H., Shukla M.S., Gerson V., Dimitrov S., Angelov D. Base excision repair of 8-oxoG in dinucleosomes. Nucleic Acids Res. 2012; 40:692–700. 21930508 10.1093/nar/gkr761 PMC3258150

[21] Charles Richard J.L., Shukla M.S., Menoni H., Ouararhni K., Roulland Y., Papin C., Ben Simon E., Kundu T., Hamiche A. et al. FACT assists base excision repair by boosting the remodeling activity of RSC. PLoS Genet. 2016; 12:e1006221. 27467129 10.1371/journal.pgen.1006221 PMC4965029

[22] Maher R.L., Marsden C.G., Averill A.M., Wallace S.S., Sweasy J.B., Pederson D.S. Human cells contain a factor that facilitates the DNA glycosylase-mediated excision of oxidized bases from occluded sites in nucleosomes. DNA Repair (Amst.). 2017; 57:91–97. 28709015 10.1016/j.dnarep.2017.06.029 PMC5569575

[23] Aslanidis C., de Jong P.J. Ligation-independent cloning of PCR products (LIC-PCR). Nucleic Acids Res. 1990; 18:6069–6074. 2235490 10.1093/nar/18.20.6069 PMC332407

[24] Luger K., Rechsteiner T.J., Richmond T.J. Expression and purification of recombinant histones and nucleosome reconstitution. Methods Mol. Biol. 1999; 119:1–16. 10804500 10.1385/1-59259-681-9:1

[25] Eccles L.J., Menoni H., Angelov D., Lomax M.E., O’Neill P. Efficient cleavage of single and clustered AP site lesions within mono-nucleosome templates by CHO-K1 nuclear extract contrasts with retardation of incision by purified APE1. DNA Repair (Amst.). 2015; 35:27–36. 26439176 10.1016/j.dnarep.2015.08.003 PMC4655832

[26] Nickson C.M., Moori P., Carter R.J., Rubbi C.P., Parsons J.L. Misregulation of DNA damage repair pathways in HPV-positive head and neck squamous cell carcinoma contributes to cellular radiosensitivity. Oncotarget. 2017; 8:29963–29975. 28415784 10.18632/oncotarget.16265 PMC5444717

[27] Edmonds M.J., Carter R.J., Nickson C.M., Williams S.C., Parsons J.L. Ubiquitylation-dependent regulation of NEIL1 by Mule and TRIM26 is required for the cellular DNA damage response. Nucleic Acids Res. 2017; 45:726–738. 27924031 10.1093/nar/gkw959 PMC5314803

[28] Williams S.C., Parsons J.L. NTH1 is a new target for ubiquitylation-dependent regulation by TRIM26 required for the cellular response to oxidative stress. Mol. Cell Biol. 2018; 38:e00616-17. 29610152 10.1128/MCB.00616-17 PMC5974432

[29] Nickson C.M., Parsons J.L. Monitoring regulation of DNA repair activities of cultured cells in-gel using the comet assay. Front. Genet. 2014; 5:232. 25076968 10.3389/fgene.2014.00232 PMC4100063

[30] Sarkar A.A., Zohn I.E. Hectd1 regulates intracellular localization and secretion of Hsp90 to control cellular behavior of the cranial mesenchyme. J. Cell Biol. 2012; 196:789–800. 22431752 10.1083/jcb.201105101 PMC3308699

[31] Meisenberg C., Tait P.S., Dianova II, Wright K., Edelmann M.J., Ternette N., Tasaki T., Kessler B.M., Parsons J.L., Kwon Y.T. et al. Ubiquitin ligase UBR3 regulates cellular levels of the essential DNA repair protein APE1 and is required for genome stability. Nucleic Acids Res. 2012; 40:701–711. 21933813 10.1093/nar/gkr744 PMC3258136

[32] Kennedy E.E., Caffrey P.J., Delaney S. Initiating base excision repair in chromatin. DNA Repair (Amst.). 2018; 71:87–92. 30170831 10.1016/j.dnarep.2018.08.011 PMC6340775

[33] Tran H., Bustos D., Yeh R., Rubinfeld B., Lam C., Shriver S., Zilberleyb I., Lee M.W., Phu L., Sarkar A.A. et al. HectD1 E3 ligase modifies adenomatous polyposis coli (APC) with polyubiquitin to promote the APC-axin interaction. J. Biol. Chem. 2013; 288:3753–3767. 23277359 10.1074/jbc.M112.415240 PMC3567630

[34] Oikonomaki M., Bady P., Hegi M.E. Ubiquitin Specific Peptidase 15 (USP15) suppresses glioblastoma cell growth via stabilization of HECTD1 E3 ligase attenuating WNT pathway activity. Oncotarget. 2017; 8:110490–110502. 29299163 10.18632/oncotarget.22798 PMC5746398

[35] Fang S., Guo H., Cheng Y., Zhou Z., Zhang W., Han B., Luo W., Wang J., Xie W., Chao J. circHECTD1 promotes the silica-induced pulmonary endothelial-mesenchymal transition via HECTD1. Cell Death Dis. 2018; 9:396. 29540674 10.1038/s41419-018-0432-1 PMC5852113

[36] Duhamel S., Goyette M.A., Thibault M.P., Filion D., Gaboury L., Cote J.F. The E3 ubiquitin ligase HectD1 suppresses EMT and metastasis by targeting the +TIP ACF7 for degradation. Cell Rep. 2018; 22:1016–1030. 29386124 10.1016/j.celrep.2017.12.096

[37] Carter R.J., Nickson C.M., Thompson J.M., Kacperek A., Hill M.A., Parsons J.L. Complex DNA damage induced by high linear energy transfer alpha-particles and protons triggers a specific cellular DNA damage response. Int. J. Radiat. Oncol. Biol. Phys. 2018; 100:776–784. 29413288 10.1016/j.ijrobp.2017.11.012 PMC5796827

[38] He Y.J., McCall C.M., Hu J., Zeng Y., Xiong Y. DDB1 functions as a linker to recruit receptor WD40 proteins to CUL4-ROC1 ubiquitin ligases. Genes Dev. 2006; 20:2949–2954. 17079684 10.1101/gad.1483206 PMC1620025

[39] Higa L.A., Wu M., Ye T., Kobayashi R., Sun H., Zhang H. CUL4-DDB1 ubiquitin ligase interacts with multiple WD40-repeat proteins and regulates histone methylation. Nat. Cell Biol. 2006; 8:1277–1283. 17041588 10.1038/ncb1490

[40] Parsons J.L., Khoronenkova S.V., Dianova II, Ternette N., Kessler B.M., Datta P.K., Dianov G.L. Phosphorylation of PNKP by ATM prevents its proteasomal degradation and enhances resistance to oxidative stress. Nucleic Acids Res. 2012; 40:11404–11415. 23042680 10.1093/nar/gks909 PMC3526271

[41] Wang H., Zhai L., Xu J., Joo H.Y., Jackson S., Erdjument-Bromage H., Tempst P., Xiong Y., Zhang Y. Histone H3 and H4 ubiquitylation by the CUL4-DDB-ROC1 ubiquitin ligase facilitates cellular response to DNA damage. Mol. Cell. 2006; 22:383–394. 16678110 10.1016/j.molcel.2006.03.035

