# Supplementary material for: HECTD1 promotes base excision repair in nucleosomes through chromatin remodelling
Source: Nucleic Acids Res. 2019 Dec 4;48(3):1301–13. doi: 10.1093/nar/gkz1129 (PMC7026656; doi:10.1093/nar/gkz1129)
Supplement: gkz1129_Supplemental_File [file gkz1129_supplemental_file.docx]

**SUPPLEMENTARY FIGURES**

**
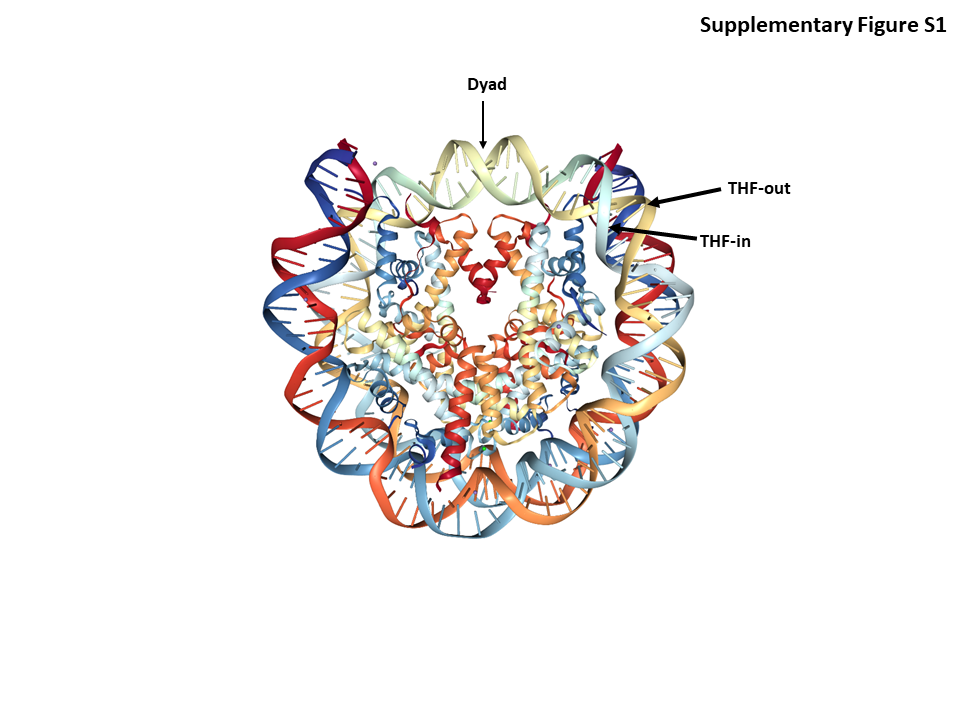
**

**Figure S1.** Structure of the nucleosome core particle composed of the 601 DNA sequence. Image was acquired from Protein Data Bank structure 3LZ0 (orientation 1), and indicated are the positions of the THF-in and THF-out sites, with the DNA backbone facing inward and outward from the histone core, respectively.

**
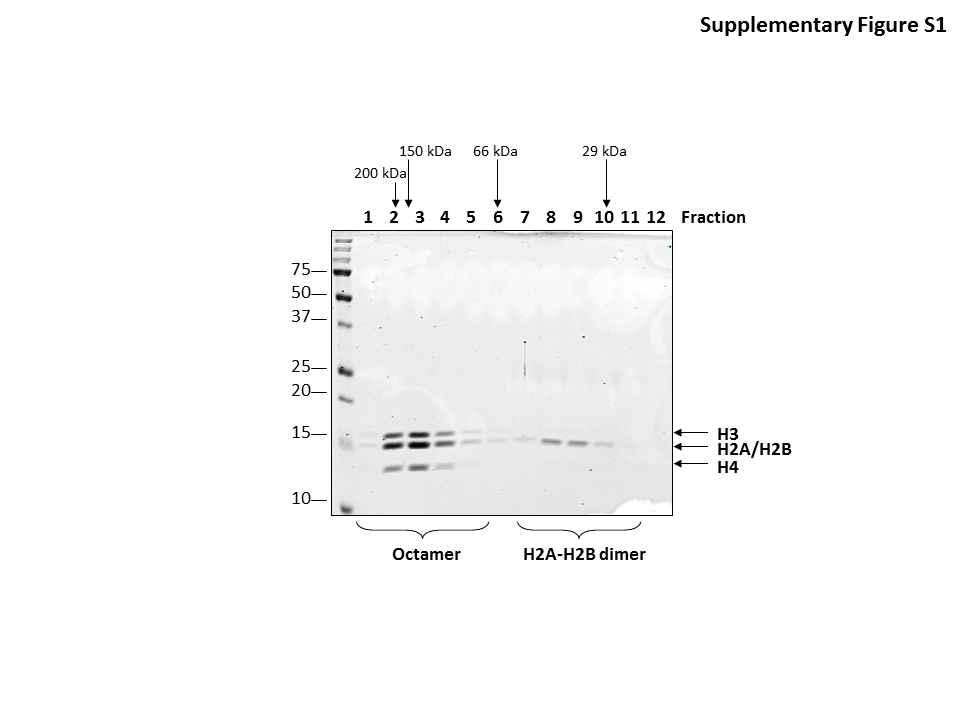
**

**Figure S2.** Purification of histone octamer by gel filtration chromatography. Histone octamer was prepared and separated by Superdex 200 10/300 GL gel filtration column, fractions were collected and analysed by 16 % SDS-PAGE and Instant blue staining. Positions of elution of known molecular weight protein standards are indicated above the fractions. Fractions 2-4 contain the majority of the histone octamer, whereas fractions 8-10 contain histone H2A-H2B dimers.


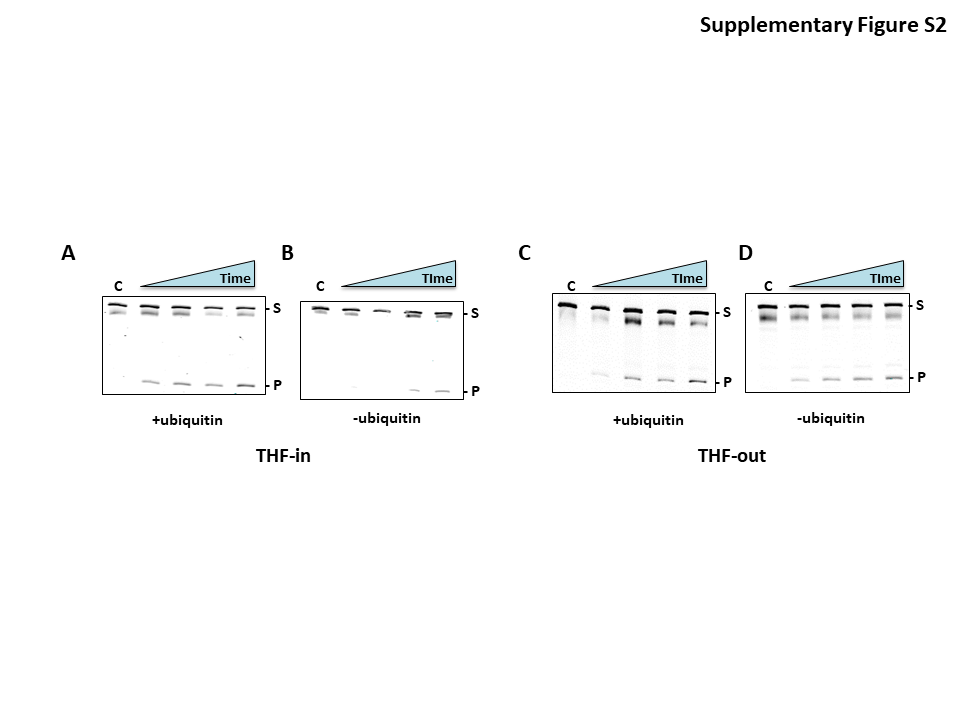


**Figure S3.** The incision of THF-in mononucleosome substrate by HeLa whole cell extract is enhanced by the addition of ubiquitin to reactions. Incision of THF-in (**A-B**) and THF-out (**C-D**) mononucleosome substrates (50 fmol) by HeLa whole cell extract (1.3 µg and 0.16 µg, respectively). Reactions were performed in the absence and presence of ubiquitin (0.6 nmol ubiquitin) and shown are representative images from the respective gels.


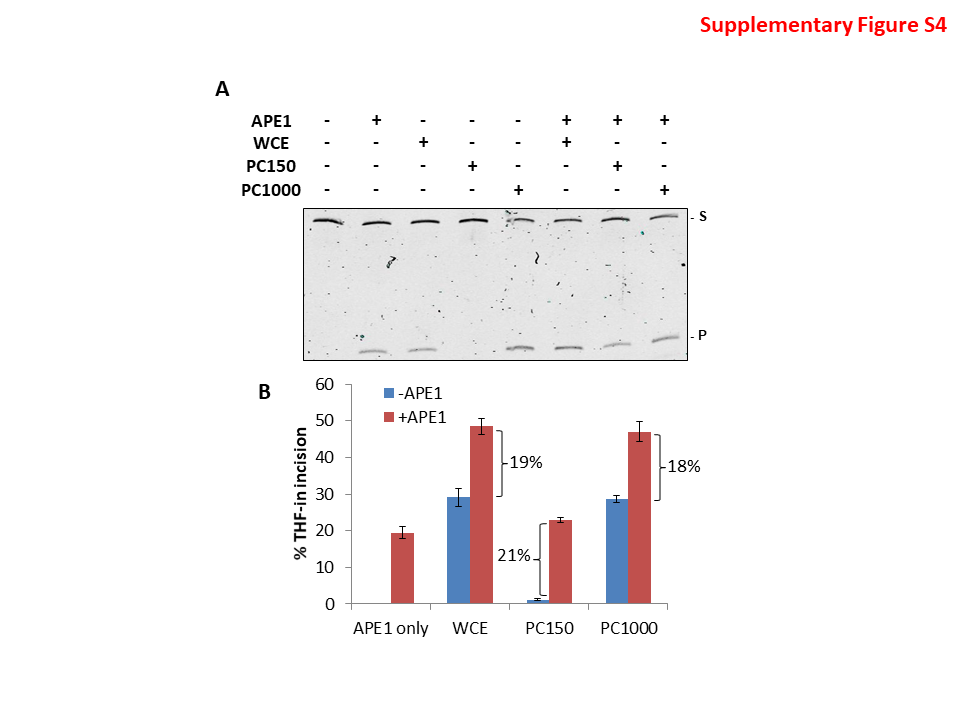


**Figure S4.** Stimulatory activities present in HeLa whole cell extract do not enhance incision of THF-in free DNA substrate by recombinant APE1**.** (**A**) Incision of the THF-in free DNA substrate (50 fmol) by WCE, PC-150 and PC-1000 (8 ng) in the absence and presence of recombinant APE1 (2 fmol). (**B**) Shown is the mean percent substrate incision ± S.D from three independent experiments, along with the difference in THF incision by the extracts in the absence and presence of recombinant APE1.

**
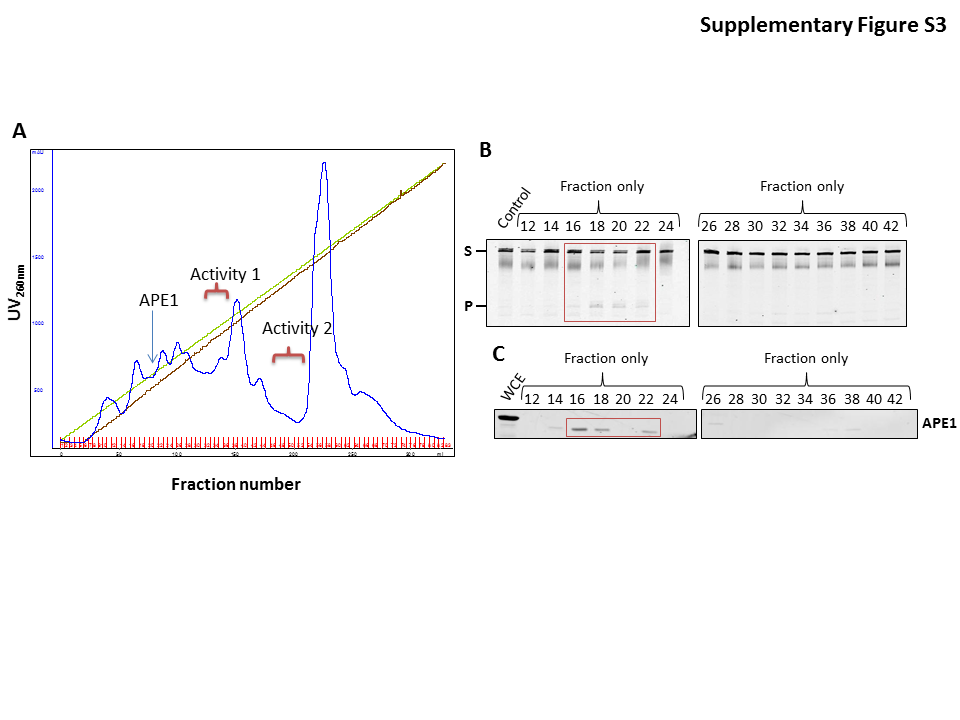
**

**Figure S5.** Identification of endogenous APE1 within fractionated HeLa cell extracts as a separate activity compared to the stimulatory activities promoting THF-in mononucleosome substrate incision. (**A**) Protein elution profile of MonoQ chromatography fractionation of HeLa PC150 fraction, demonstrating the positions of elution of endogenous APE1, and the two activities (Activity 1 and 2) enhancing incision of the THF-in mononucleosome substrate by recombinant APE1. (**B**) Representative image of the activity of fractions 12-42 only against the THF-in mononucleosome substrate along with a control reaction containing substrate only. (**C**) Fractions were immunoblotted using APE1 antibodies, along with a HeLa whole cell extract (WCE) control.

**
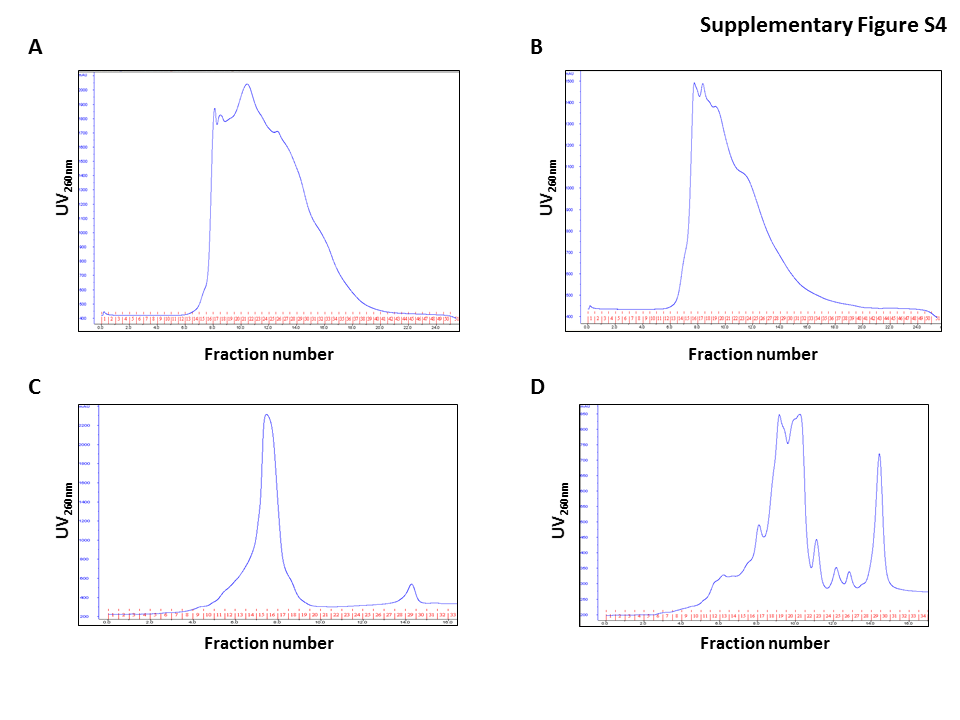
**

**Figure S6.** Protein elution profiles of fractionated HeLa cell extracts containing stimulatory activities promoting THF-in mononucleosome substrate incision by recombinant APE1. (**A-B**) Superdex 200 chromatography and (**C-D**) final MonoQ fractionation of HeLa cell extracts. Protein fractions were successively generated by the two chromatography columns using fractions combined from the first MonoQ chromatography containing Activity 1 (**A** and **C**) and Activity 2 (**B** and **D**).

**
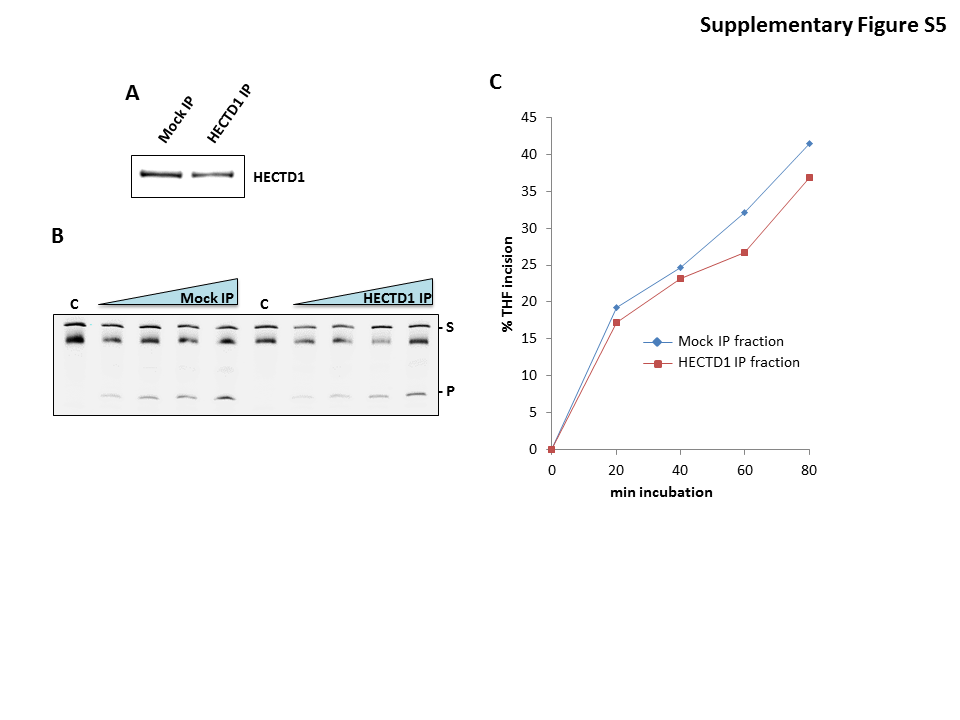
**

**Figure S7.** HECTD1 could not be successfully immunodepleted from active HeLa cell extract chromatography fractions. (**A**) An active fraction (Fraction 16) generated from the final MonoQ chromatography stage of purification of stimulatory Activity 1 was immunodepleted using HECTD1 antibodies bound to protein A magnetic beads, or using beads only (Mock IP). Levels of HECTD1 were analysed by immunoblotting. (**B**) Representative image demonstrating stimulation of incision of THF-in mononucleosome substrate (50 fmol) by recombinant APE1 (60 fmol) by mock and HECTD1 immunodepleted fraction over a time course. Control reactions (C) contained substrate only. (**C**) Shown is the mean percent substrate incision from a single experiment, normalised to 0 after subtraction of the incision observed with recombinant APE1 only (~20 %), demonstrating a slight reduction in stimulatory activity of the HECTD1 immunodepleted fraction.

**
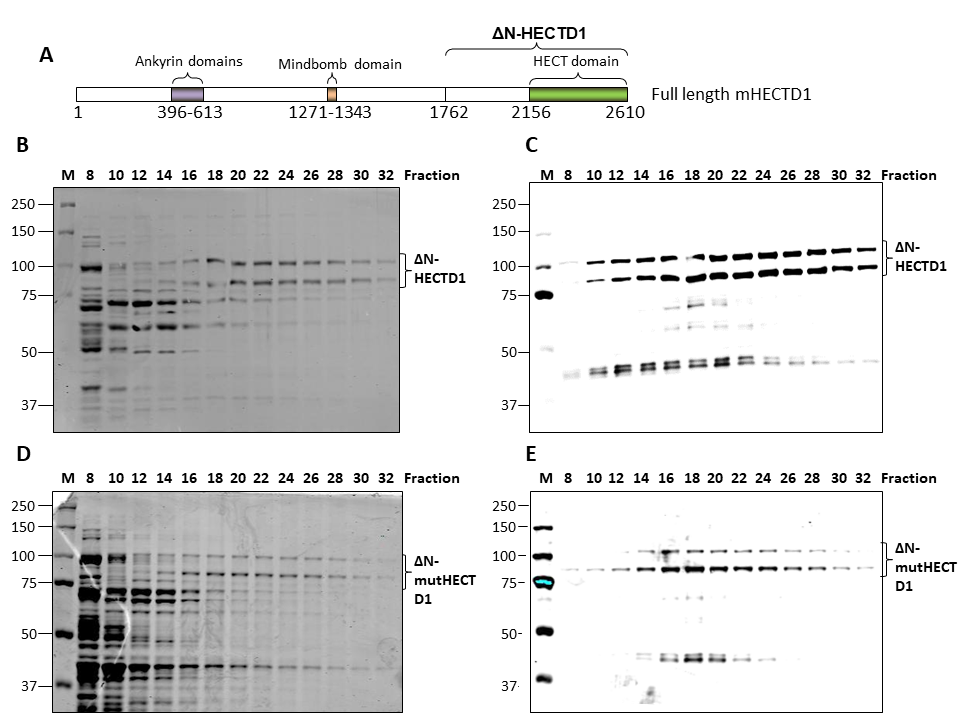
**

**Figure S8.** Purification of wild type and mutant HECTD1 recombinant proteins by histag chromatography**.** (**A**) Diagrammatic representation of the mHECTD1 protein highlighting the major domains (ankyrin, Mindbomb and HECT) present within the protein. A C-terminal truncation of murine HECTD1 (amino acids 1762-2612; ΔN-HECTD1) and a catalytically inactive E3 ligase mutant (C2579G; ΔN-mutHECTD1) were overexpressed in bacterial cells, a cell lysate was generated which was then separated using His-tag chromatography incorporating an imidazole elution gradient. (**B** and **D**) Total protein in the fractions as revealed by Instant Blue staining and (**C** and **E**) detection of the HECTD1 proteins by immunoblotting using histag antibodies. M refers to prestained protein markers.


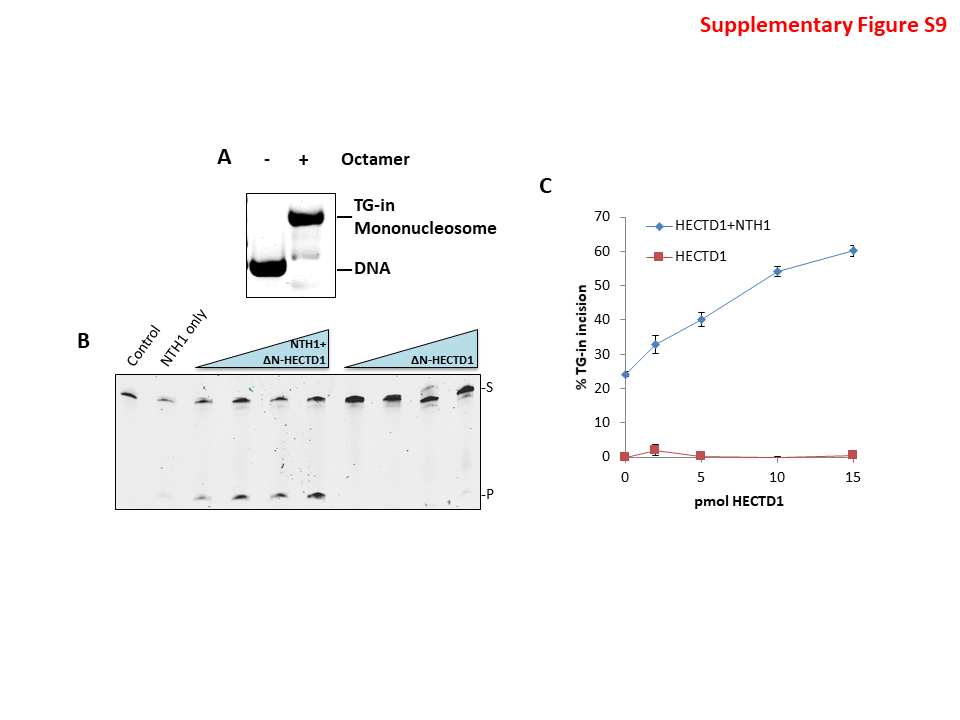


**Figure S9.** HECTD1 promotes excision of TG-in mononucleosome substrate by recombinant NTH1 *in vitro*. (**A**) Mononucleosome DNA substrate containing thymine glycol with the backbone inwardly facing (TG-in) was prepared by salt dialysis following incubation of the DNA substrate with histone octamer, and analysed by agarose gel electrophoresis. (**B**-**C**) Stimulation of NTH1-dependent (1.5 pmol) incision of the TG-in mononucleosome substrate (50 fmol) by increasing amounts of wild type ΔN-HECTD1. (**C**) Shown is the mean percent substrate incision ± S.D. from three independent experiments by ΔN-HECTD1 in the absence and presence of NTH1. The respective control reactions were performed in the absence of any protein (Control) and with recombinant NTH1 only.

**
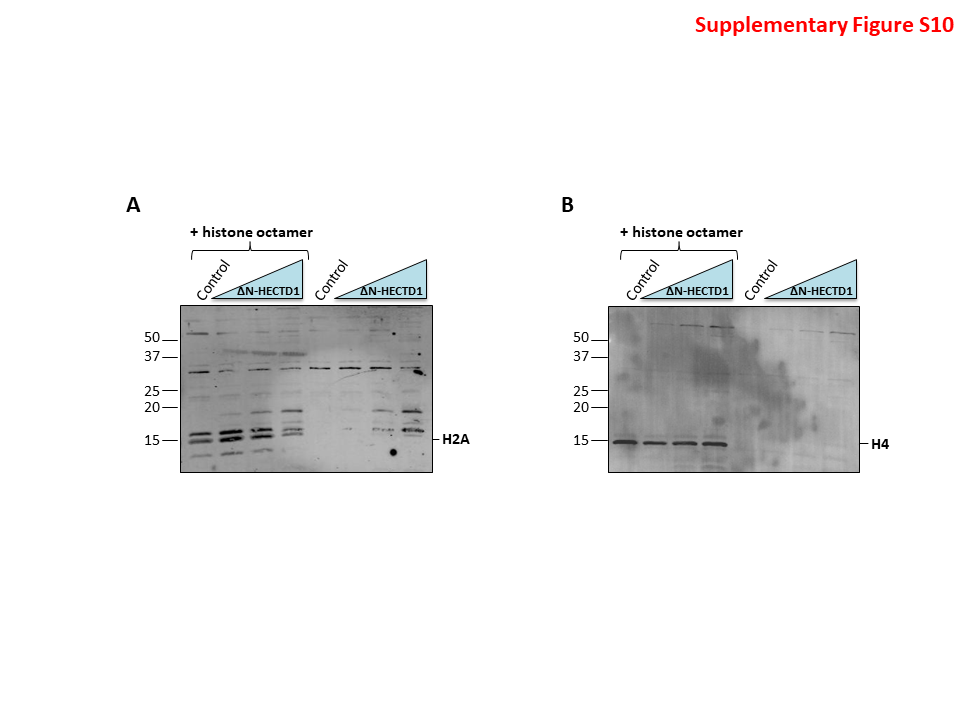
**

**Figure S10.** HECTD1 does not appear to stimulate *in vitro* ubiquitylation of histones H2A or H4 when present within the histone octamer**.** *In vitro* ubiquitylation assays containing histone octamer (2 pmol) were incubated in the presence of increasing amounts of ΔN-HECTD1 (2.8-14.1 pmol). Samples were separated by 16 % SDS-PAGE and analysed by immunoblotting using (**A**) histone H2A or (**B**) H4 antibodies. The respective control reactions (Control) were performed in the absence of any ΔN-HECD1.

**
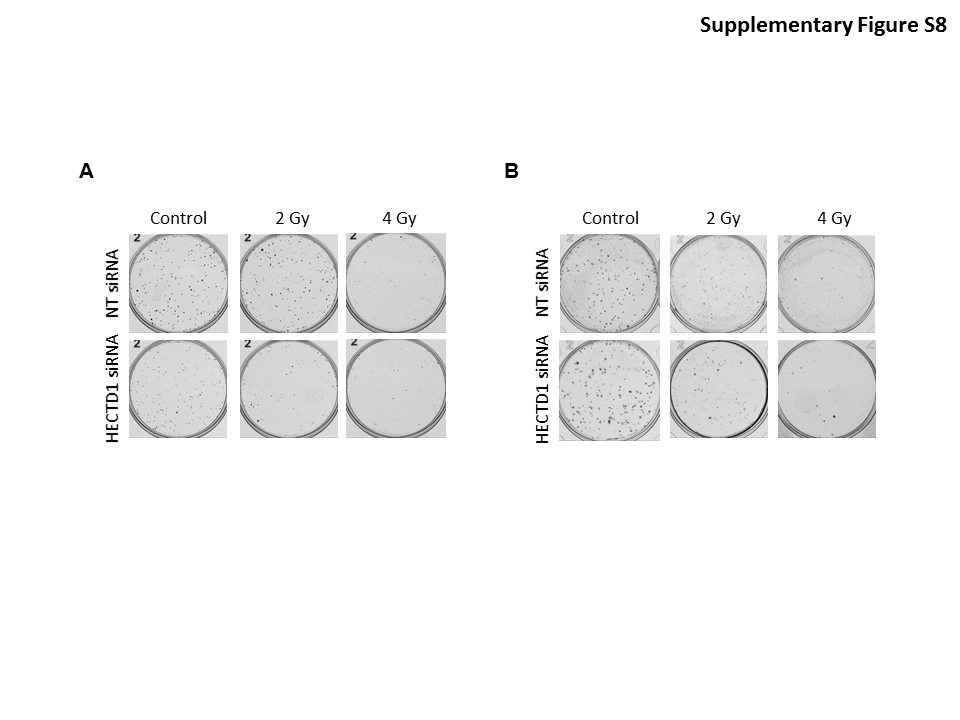
**

**Figure S11.** A reduction in the protein levels of HECTD1 enhances cellular radiosensitivity. (**A**) WI-38 or (**B**) HeLa cells were treated with 40 nM non-targeting (NT) control siRNA or HECTD1 siRNA for 48 h. Cells were then unirradiated (Control) or irradiated with 2 or 4 Gy x-ray irradiation, trypsinised, counted and a defined number plated into 6 well plates. Increasing cell numbers were used for increasing doses of x-ray irradiation to account for cellular plating efficiencies. Colonies were allowed to grow for 7-10 days prior to staining.


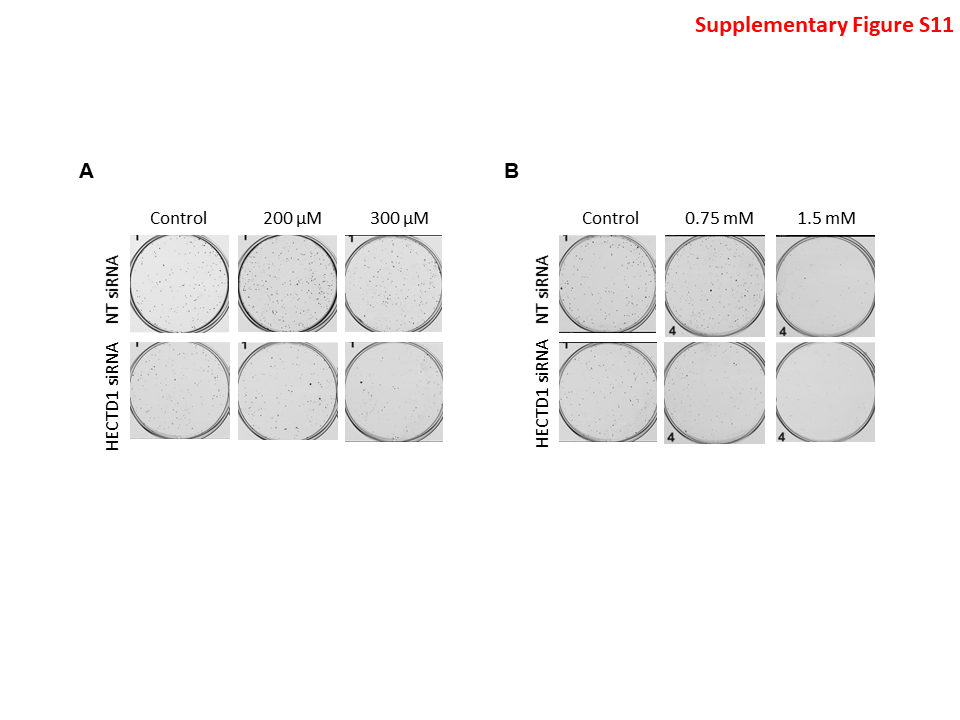


**Figure S12.** A reduction in the protein levels of HECTD1 enhances cellular sensitivity to hydrogen peroxide and methylmethanesulfonate. WI-38 cells were treated with 40 nM non-targeting (NT) control siRNA or HECTD1 siRNA for 48 h. Cells were then either untreated (Control) or treated with (**A**) 200 or 300 µM H_2_O_2_ or (**B**) 0.75 or 1.5 mM MMS, trypsinised, counted and a defined number plated into 6 well plates. Increasing cell numbers were used for increasing doses of the DNA damaging agent to account for cellular plating efficiencies. Colonies were allowed to grow for 7-10 days prior to staining.
